# ZnO-modified activated carbon derived from rambutan (*Nephelium lappaceum*) peel and seeds for efficient methylene blue removal: adsorption mechanism and artificial neural network modeling

**DOI:** 10.1039/d6ra01824f

**Published:** 2026-04-10

**Authors:** Tra Huong Do, Thị Nguyet Hua, Manh Nhuong Chu, Thi Hien Lan Nguyen, Truong Xuan Vương

**Affiliations:** a Faculty of Chemistry, Thai Nguyen University of Education No. 20 Luong Ngoc Quyen Street Thai Nguyen City 24000 Vietnam; b Faculty of Natural Sciences and Technology, TNU-University of Science Tan Thinh Ward Thai Nguyen City 24000 Vietnam xuanvt@tnus.edu.vn

## Abstract

A ZnO-carbon hybrid (ZnO-ACRPS) was constructed through a dual-biomass route combining rambutan peel and seeds as structurally complementary carbon sources. Hydrothermal carbonization, subsequent pyrolysis, and controlled ZnO deposition converted the lignin-dominated peel framework and heteroatom-rich seed fractions into a turbostratic carbon network with regulated disorder and hierarchical mesoporosity. The composite reached a specific surface area of 575.81 m^2^ g^−1^ with an average pore diameter of 3.23 nm. Wurtzite ZnO nanocrystals (10–40 nm) were uniformly dispersed throughout the matrix, introducing accessible Lewis acidic centers within the pore architecture. Interfacial compositional tuning influenced adsorption energetics. Methylene blue uptake was better described by the Langmuir model, yielding a maximum capacity of 82.95 mg g^−1^ at pH 7, and followed pseudo-second-order kinetics. Mass transfer was governed by coupled film diffusion and intraparticle diffusion steps. Negative Gibbs free energy values indicate spontaneous uptake, whereas an enthalpy change near 68 kJ mol^−1^ suggests an endothermic contribution consistent with strong surface interactions rather than purely weak physisorption. Dye binding can be attributed to π–π interactions between graphitic domains and aromatic rings, electrostatic attraction governed by pH_p_zc, and may involve ZnO-centered Lewis acid–base interactions. A feedforward artificial neural network (5–13–1) demonstrated good predictive performance and agreement with experimental results (*R*_test_^2^ = 0.921; RMSE = 5.86). Sensitivity analysis identified temperature and adsorbent dosage as the most influential factors, consistent with thermally activated adsorption and active-site accessibility inferred from surface and thermodynamic analyses. Regeneration trials indicated maintained performance during initial cycles, followed by a noticeable decline upon repeated use, while structural features remained preserved. Dual-precursor compositional control and ANN-assisted analysis establish a framework for understanding structure–performance relationships in ZnO-carbon systems, while providing a basis for future evaluation under more complex wastewater conditions.

## Abstract 2

A ZnO-carbon hybrid (ZnO-ACRPS) was constructed through a dual-biomass route combining rambutan peel and seeds as structurally complementary carbon sources.

## Graphical abstract

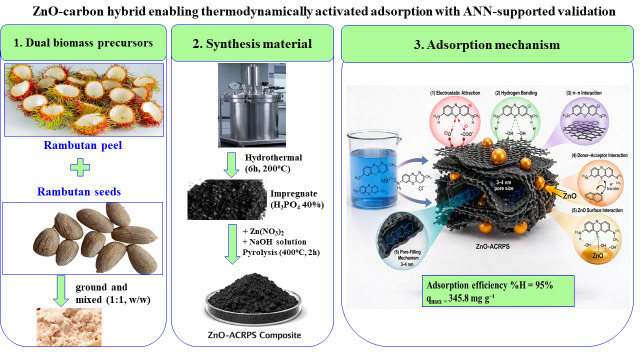

## 1. Introduction

The rapid expansion of textile, printing, paper, paint, and pharmaceutical industries has substantially increased the discharge of synthetic dyes into aquatic environments.^1,2^ Many dyes possess stable aromatic structures and low biodegradability, leading to persistent coloration, reduced light penetration, inhibition of photosynthesis, and depletion of dissolved oxygen in receiving waters.^3,4^ Certain dye compounds may also bioaccumulate and exert toxic effects on aquatic organisms and humans.

Methylene blue (MB), a cationic heterocyclic aromatic dye widely used in industry and aquaculture, is frequently detected in wastewater streams.^1,2^ Its conjugated structure confers high chemical stability and resistance to natural degradation. Prolonged exposure has been associated with physiological disorders and growth inhibition in aquatic species,^5,6^ and its discharge is therefore regulated in many countries.^7^

MB is also one of the most extensively studied model dyes in adsorption research due to its well-defined structure, stability, and ease of spectroscopic detection. Numerous adsorbents, including activated carbons, metal oxides, and composite materials, have been reported for MB removal, as summarized in several comprehensive studies.^8,9^ In many cases, higher adsorption capacities than those obtained in the present study have been reported,^10^ highlighting that MB adsorption is a well-established and widely explored research area.

Among available treatment technologies, including advanced oxidation processes,^11^ membrane filtration, coagulation–flocculation,^12^ and biological treatment,^13^ adsorption remains one of the most practical approaches due to operational simplicity, minimal secondary pollution, and regeneration potential. In particular, biomass-derived carbon materials have attracted increasing attention within circular economy frameworks. Agricultural residues can be transformed into biochar^14,15^ and activated carbon,^4,16,17^ generating porous structures with high surface area and oxygen-containing functional groups suitable for dye adsorption.^3,4,18^ However, adsorption performance strongly depends on pore architecture and surface chemistry, necessitating rational structural modification.

Various adsorbent materials, including biochar, cyclodextrin-based materials, and chitosan-derived adsorbents, have been widely investigated for wastewater treatment. Biochar offers a low-cost and sustainable platform; however, its adsorption performance is often limited by insufficient active sites and surface functionality unless modified.^19^ Cyclodextrin-based adsorbents exhibit selective host–guest inclusion properties but may be less effective for non-specific adsorption of diverse pollutants. Chitosan-based materials provide abundant functional groups (*e.g.*, –NH_2_) and strong affinity toward certain contaminants, although their chemical stability and performance can be sensitive to pH and environmental conditions.^20^

In this context, ZnO-modified activated carbon has emerged as a promising approach as it combines the high surface area and porosity of activated carbon with the surface reactivity and catalytic properties of ZnO nanoparticles. The incorporation of ZnO can enhance adsorption performance by introducing additional active sites, improving surface charge properties, and enabling synergistic adsorption mechanisms.^21^ Therefore, ZnO-modified activated carbon represents an effective hybrid system for efficient removal of organic pollutants from aqueous environments.

While natural adsorbents such as biochar and chitosan-based materials offer advantages in terms of low cost and sustainability, their adsorption performance is often limited by restricted active-site diversity and less controllable surface properties. In contrast, hybrid systems such as ZnO–carbon composites enable additional interaction pathways and tunable interfacial chemistry. Therefore, the objective of this study is not to replace conventional natural adsorbents, but to provide mechanistic insight into how compositional design and interfacial structure influence adsorption behavior, supported by data-driven modeling.

However, despite these advantages, many studies primarily emphasize adsorption capacity and conventional isotherm fitting, while comparatively less attention has been given to understanding the underlying structure–property relationships and interfacial interactions that govern adsorption behavior.^8,9^ Recent studies have highlighted that adsorption performance is strongly influenced by surface chemistry, pore structure, and interfacial effects, which are often insufficiently addressed in capacity-driven investigations.^22,23^ These limitations highlight the need for developing hybrid systems with controlled composition and interfacial structure using sustainable biomass precursors.

Rambutan (*Nephelium lappaceum*), extensively cultivated in Southeast Asia, generates substantial peel and seed residues during processing.^3,4^ These lignocellulosic materials, rich in cellulose, hemicellulose, lignin, polyphenols, and flavonoids,^24–26^ are promising carbon precursors. The aromatic-rich lignin framework of the peel provides a stable carbon scaffold, while the lipid and protein components of the seeds introduce endogenous heteroatoms, potentially leading to a self-doped turbostratic structure that enhances the anchoring of ZnO nanoparticles. Previous studies have utilized either rambutan peel^26,27^ or seeds^25,28,29^ to prepare activated carbons with encouraging adsorption performance.^3,4,25,29^ Nevertheless, the combined utilization of both peel and seeds to engineer hierarchical porosity and synergistic surface chemistry remains largely unexplored.^4^ Moreover, pristine biomass-derived carbons may exhibit limited active site density and restricted intraparticle diffusion compared with modified composite systems.^30^

Incorporating zinc oxide (ZnO) into carbon matrices represents an effective strategy to enhance surface functionality and introduce additional adsorption sites.^31,32^ ZnO possesses pH-dependent surface charge and Lewis acidic Zn^2+^ centers that enable electrostatic attraction and coordination interactions with dye molecules. When uniformly dispersed within porous carbon frameworks, ZnO nanoparticles can modulate pore structure, interfacial bonding, and active-site accessibility,^33^ often improving adsorption capacity and structural stability.^34,35^ However, in many reported ZnO-carbon systems, the relationship between ZnO dispersion, ZnO-carbon interfacial structure, and adsorption efficiency remains poorly understood.^34^ This knowledge gap is particularly critical for dual-biomass-derived composites, where precursor interactions may significantly influence microstructural evolution and active-site distribution.

Moreover, adsorption in hybrid oxide–carbon systems is governed by coupled nonlinear effects of pH, adsorbent dosage, temperature, initial concentration, and contact time. Conventional kinetic–isotherm modeling and linear regression approaches may not fully capture these multivariate interactions.^36,37^ Artificial neural networks (ANNs) provide data-driven predictive capability without imposing strict thermodynamic assumptions. They also allow quantification of variable importance *via* sensitivity analysis.^38,39^ Nevertheless, their integration with physicochemical interpretation to support mechanistic understanding remains relatively underexplored.

In this context, the present study does not aim to achieve the highest adsorption capacity, but rather to elucidate the relationship between compositional design, interfacial structure, and adsorption behavior. A ZnO-decorated activated carbon composite was synthesized from a dual-biomass system (rambutan peel and seed) to enable controlled structural and chemical tuning. Furthermore, ANN modeling was incorporated to provide data-supported insight into adsorption trends and key governing variables.

Therefore, the novelty of this work lies in (i) the dual-biomass strategy enabling compositional and structural control, (ii) the investigation of ZnO-carbon interfacial contributions to adsorption behavior, and (iii) the integration of ANN-based analysis to support physicochemical interpretation. This integrated experimental–computational framework provides deeper insight into structure–performance relationships and supports the rational design of sustainable ZnO-carbon hybrid adsorbents for dye-contaminated wastewater systems.

## 2. Materials and methods

### 2.1. Materials

#### 2.1.1. Biomass precursor

Rambutan peel and seed were used as biomass precursors for the synthesis of carbon materials. The raw materials were collected from local fresh fruit markets.

#### 2.1.2. Chemicals and reagents

All chemicals used in this study were of analytical grade and were used without further purification.

• Zinc salt (Zn^2+^ precursor): zinc nitrate hexahydrate (Zn(NO_3_)_2_·6H_2_O, ≥99%, Sigma-Aldrich) was employed as a modification agent to promote pore development and/or facilitate the dispersion of metal oxide phases on the carbon surface.

• Phosphoric acid (H_3_PO_4_, 40%) was used as a chemical activating agent.

• Sodium bicarbonate (NaHCO_3_) was applied as a dilute solution to neutralize residual acid after activation and to adjust the material pH to near neutrality (pH ≈ 7).

• Double-distilled water was used throughout the washing and solution preparation steps to minimize the influence of foreign ions on the structure and surface properties of the materials.

### 2.2. Preparation of activated carbon

Activated carbon was synthesized from rambutan peel and seed through a combined process of hydrothermal treatment, chemical activation, and high-temperature pyrolysis.

#### 2.2.1. Raw material pretreatment

Fresh rambutan peel and seed were washed several times with tap water and then rinsed with distilled water to remove dust, mechanical impurities, and surface-adhered organic compounds. The materials were dried at 80 °C for 24 h to remove free moisture.

The dried biomass was mechanically ground and sieved to obtain particles with a uniform size range of 2–5 mm. Controlling particle size ensured homogeneity and reproducibility in subsequent processing steps.

#### 2.2.2. Carbonization conditions

The pretreated biomass was dispersed in distilled water at a mass-to-volume ratio of 1 : 10 (g mL^−1^). The suspension was transferred into a Teflon-lined autoclave, sealed, and heated at 200 °C for 6 h. After hydrothermal treatment, the reactor was allowed to cool naturally to room temperature. The obtained solid product (hydrochar) was separated, thoroughly washed with distilled water to remove soluble by-products, and dried at 105 °C to constant weight.

#### 2.2.3. Chemical activation

Chemical activation was performed using 40% phosphoric acid (H_3_PO_4_). The hydrochar was impregnated with H_3_PO_4_ at a mass ratio of H_3_PO_4_ : hydrochar = 3 : 1. The mixture was gently stirred and kept at room temperature for 24 h to ensure thorough penetration of the activating agent into the carbon structure. H_3_PO_4_ acts as a dehydrating agent and crosslinking catalyst, promoting the formation of a well-developed porous structure during subsequent thermal treatment.

#### 2.2.4. Washing and drying

After impregnation, the material was washed with dilute NaHCO_3_ solution to neutralize residual acid. Washing was continued until the filtrate reached approximately neutral pH (≈7). The sample was then repeatedly rinsed with distilled water to completely remove salts and residual impurities, followed by drying to constant weight. The obtained activated carbon was stored in a sealed desiccator to prevent moisture adsorption and environmental contamination. The final material was denoted as ACRPS.

### 2.3. ZnO modification procedure

The ZnO-ACRPS composite was synthesized *via* an impregnation-*in situ* precipitation method followed by thermal treatment.

First, 1.5 g of Zn(NO_3_)_2_·6H_2_O was completely dissolved in 100 mL of distilled water to obtain a homogeneous Zn^2+^ precursor solution. This solution was slowly added to a flask containing 5 g of ACRPS under continuous magnetic stirring for 4 h at room temperature to ensure uniform distribution of Zn^2+^ ions on the surface and within the pore system of the carbon matrix.

After impregnation, 1 M NaOH solution was added dropwise to the reaction system until the pH reached approximately 10–11, creating an alkaline environment favorable for the precipitation of Zn(OH)_2_. The mixture was further stirred for 2 h to ensure complete precipitation and effective anchoring of Zn(OH)_2_ onto the ACRPS surface.

The resulting product was filtered and washed repeatedly with distilled water until the filtrate reached neutral pH to remove excess ions and soluble impurities. The sample was dried at 80 °C for 12 h to remove moisture.

Subsequently, calcination was performed in a furnace at 400 °C for 2 h with a heating rate of 5 °C min^−1^ under a continuous nitrogen atmosphere (N_2_ flow rate: 100 mL min^−1^). The inert environment prevented oxidative degradation of the carbon framework during pyrolysis.

This thermal treatment simultaneously converted Zn(OH)_2_ to ZnO and promoted the fixation and dispersion of ZnO particles on the carbon matrix, forming the ZnO-biochar composite. The obtained material was denoted as ZnO-ACRPS.

The theoretical ZnO loading in the composite was estimated to be approximately 7.6 wt%.

**Fig. 1:**
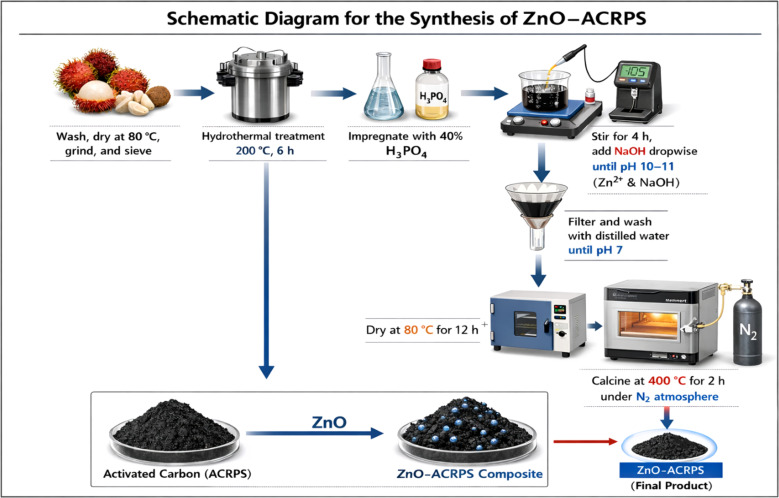
Schematic diagram for the synthesis of ZnO-ACRPS.

The overall preparation procedure is illustrated schematically as Fig. 1.

### 2.4. Characterization techniques

#### 2.4.1. Specific surface area and pore size distribution (BET)

The specific surface area and pore characteristics of ZnO-ACRPS were determined by N_2_ adsorption–desorption measurements at 77 K using a TriStar II 3020 analyzer (Micromeritics, USA). Prior to analysis, samples were degassed under vacuum at an appropriate temperature to remove moisture and physically adsorbed species from the surface.

The specific surface area was calculated using the Brunauer–Emmett–Teller (BET) model within the appropriate relative pressure range. The total pore volume was estimated from the amount of nitrogen adsorbed at *p*/*p*_0_ ≈ 0.99, while the pore size distribution was derived from the desorption branch using the BJH method. The adsorption isotherms were classified according to IUPAC standards to evaluate the pore structure of the material.

#### 2.4.2. X-ray diffraction (XRD)

The crystalline structure and phase composition of the synthesized materials were examined by X-ray diffraction (XRD) using an Equinox 5000 diffractometer (Thermo Scientific, France) with Cu Kα radiation (*λ* = 1.5406 Å). Diffraction patterns were recorded over a 2*θ* range of 10–80°, with a step size of 0.02° and a scanning rate of 2° min^−1^ to ensure reliable phase identification.

Phase identification was performed by comparison with the Powder Diffraction File (PDF) database to confirm the formation of hexagonal wurtzite ZnO and to evaluate the structural ordering of the carbon matrix. The interplanar spacing (*d*-spacing) was calculated using Bragg's law:

1 *nλ* = 2*d* sin *θ*

where *n* is the diffraction order, *λ* is the X-ray wavelength, *d* is the lattice spacing, and *θ* is the diffraction angle.

Peak broadening was qualitatively analyzed to assess crystallinity evolution and structural stability before and after regeneration cycles. The presence or absence of characteristic ZnO reflections after reuse provided evidence of phase stability and resistance to sintering or leaching during adsorption–desorption processes.

#### 2.4.3. Scanning and transmission electron microscopy (SEM/TEM)

The surface morphology and microstructure of the materials before and after modification were examined using scanning electron microscopy (SEM, JSM-6510LV, JEOL, Japan). Elemental composition was analyzed by energy-dispersive X-ray spectroscopy (EDS) integrated into the SEM system to confirm the presence and distribution of Zn on the carbon matrix.

The nanostructure and dispersion of ZnO particles on ACRPS were further observed by transmission electron microscopy (TEM). ZnO particle size was estimated from TEM images, and the anchoring behavior of metal oxide particles on the carbon framework was evaluated.

#### 2.4.4. Fourier transform infrared spectroscopy (FTIR)

Surface functional groups before and after adsorption were analyzed using Fourier transform infrared spectroscopy (FTIR) with a Nicolet Nexus 670 spectrometer (Thermo Scientific, USA). Spectra were recorded in the wavenumber range of 4000–400 cm^−1^.

Characteristic absorption bands were assigned to oxygen-containing functional groups (–OH, C=O, C–O), aromatic structures (C=C), and Zn–O vibrations. Changes in peak position or intensity after adsorption were used to evaluate the involvement of surface functional groups in interactions with MB molecules.

#### 2.4.5. Determination of pH_pzc_

The point of zero charge (pH_pzc_) of ZnO-ACRPS was determined using the pH drift method. A 0.01 M NaCl solution was used as the background electrolyte to maintain constant ionic strength. A series of 50 mL NaCl solutions (0.01 M) were adjusted to initial pH values (pH_0_) between 2 and 11 using 0.1 M HCl or 0.1 M NaOH. Subsequently, 0.05 g of ZnO-ACRPS was added to each flask. The suspensions were sealed and shaken at 150 rpm for 24 h at 25 ± 1 °C to reach equilibrium. After equilibration, the final pH (pH_f_) was measured using a calibrated pH meter. The change in pH (ΔpH) was calculated as:

2 ΔpH = pHf − pH_0_

The pH_pzc_ was determined from the intersection point of the ΔpH *versus* pH_0_ plot with the horizontal axis (ΔpH = 0). The pH_pzc_ value was used to evaluate the surface charge properties of the material and to predict electrostatic interactions between the adsorbent and MB molecules at different pH conditions.

### 2.5. Batch adsorption experiments

Batch adsorption experiments were conducted to evaluate the influence of operational parameters on methylene blue (MB) removal by ZnO-ACRPS. All experiments were performed in 100 mL conical flasks under static adsorption conditions. After adsorption, samples were centrifuged at 4000 rpm for 15 min to separate the solid phase, and the residual MB concentration was determined by UV-Vis spectrophotometry at its characteristic wavelength.

Each experiment was conducted in triplicate under identical conditions, and results are presented as mean values.

The removal efficiency (%H) and adsorption capacity at time *t* (*q*_*t*_, mg g^−1^) were calculated as follows:3
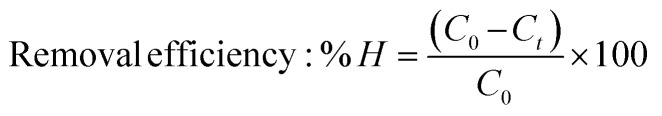
4
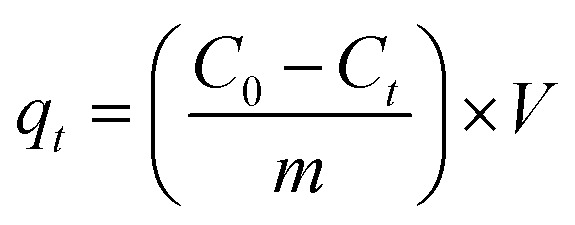
where *C*_0_ (mg L^−1^) is the initial MB concentration, *C*_*t*_ (mg L^−1^) is the concentration at time *t*, *V* (L) is the solution volume, and *m* (g) is the adsorbent mass.

MB concentration was determined using a UV-Vis spectrophotometer at *λ*_max_ = 664 nm. A calibration curve was established within the linear range of 1–10 mg L^−1^ (*R*^2^ > 0.9974), ensuring accurate quantification. Detailed calibration data are provided in Fig. S1 (see (SI)).

#### 2.5.1. Comparative adsorption performance of ZnO-ACRPS and ACRPS

The adsorption performance of ACRPS and ZnO-ACRPS toward MB was evaluated under identical batch conditions. Briefly, 0.05 g of each material was added to 25 mL of 50 mg L^−1^ MB solution (pH 7). The pH was adjusted using 0.1 M NaOH or 0.1 M HNO_3_.

The suspensions were shaken at 175 rpm for 120 min at 25 ± 1 °C to ensure equilibrium. After centrifugation, the residual MB concentration was measured by UV-Vis spectrophotometry.

ZnO-ACRPS achieved a removal efficiency of 95.58%, significantly higher than ACRPS (48.97%) (Fig. S1, SI). The improved performance is attributed to ZnO modification, which increased the number and diversity of active adsorption sites, enhanced surface area, and improved surface chemistry. Dispersed ZnO particles likely introduced additional electrostatic and surface interaction sites, thereby enhancing dye removal. Based on these results, ZnO-ACRPS was selected for subsequent adsorption studies.

#### 2.5.2. Effect of pH

The effect of pH was investigated within the range of 3–9. Specifically, 0.05 g of ZnO-ACRPS was added to 25 mL of MB solution with an initial concentration of 50 mg L^−1^. The pH values were adjusted using either 0.1 M HCl or 0.1 M NaOH prior to the adsorption process. The samples were agitated at 175 rpm for 120 min at 25 ± 1 °C.

#### 2.5.3. Effect of contact time

Adsorption kinetics were studied by adding 0.05 g of the material into 25 mL of MB solutions with initial concentrations of 50, 70, and 90 mg L^−1^ (at pH 7). The adsorption process was conducted over various time intervals (30–210 min) at 25 ± 1 °C with a shaking speed of 175 rpm. Samples were collected at predetermined time points to analyze the residual MB concentration.

#### 2.5.4. Effect of initial concentration

The influence of initial concentration was examined in the range of 50–550 mg L^−1^. In each experiment, 0.05 g of ZnO-ACRPS was introduced into 25 mL of MB solution at pH 7. The mixtures were shaken for 120 min at 25 ± 1 °C and 175 rpm to ensure that adsorption equilibrium was reached.

#### 2.5.5. Effect of adsorbent dosage

The impact of adsorbent dosage was evaluated by varying the mass of ZnO-ACRPS from 0.01 to 0.07 g in 25 mL of 50 mg L^−1^ MB^−1^ solution (pH 7). The adsorption procedure was maintained for 120 min at 25 ± 1 °C and a stirring speed of 175 rpm.

#### 2.5.6. Effect of agitation speed

A series of 100 mL Erlenmeyer flasks were prepared. Each flask contained 0.05 g of ZnO-ACRPS and 25 mL of MB solution with an accurately determined initial concentration of 50.00 mg L^−1^. The solution pH was adjusted and maintained at pH 7 using 0.1 M NaOH and 0.1 M HNO_3_ solutions. The adsorption process was carried out on a thermostatic magnetic stirrer for 120 minutes. The stirring speeds were set at 35, 175, and 300 rpm. All experiments were conducted at room temperature (25 ± 1 °C). After adsorption, the suspensions were centrifuged to separate the solid phase.The residual MB concentration in the supernatant was then determined.

#### 2.5.7. Effect of temperature

The effect of temperature was investigated at 303, 313, and 323 K. In each trial, 0.05 g of ZnO-ACRPS was added to 25 mL of 150 mg L^−1^ MB^−1^ solution at pH 7. The adsorption process was carried out for 120 min with a shaking speed of 175 rpm. The obtained data were utilized to evaluate the thermodynamic nature of the adsorption process.

### 2.6. Adsorption modeling

Adsorption Adsorption data were systematically analyzed using kinetic, isotherm, and thermodynamic models to elucidate the underlying mechanism of methylene blue (MB) uptake onto ZnO-ACRPS. Model parameters were estimated *via* non-linear regression by minimizing the sum of squared errors (SSE), ensuring accurate representation of the experimental data.

To comprehensively evaluate the goodness-of-fit and model reliability, multiple statistical criteria were employed, including the chi-square statistic (*χ*^2^), coefficient of determination (*R*^2^), root mean square error (RMSE), and akaike information criterion (AIC). While *R*^2^ provides a measure of the correlation between experimental and predicted values, *χ*^2^ and RMSE quantify the deviation and dispersion of residuals, offering insight into model accuracy. In addition, AIC was used as an information-theoretic criterion to assess model quality by balancing goodness-of-fit and model complexity, thereby enabling robust comparison and ranking among competing models.

This multi-criteria evaluation approach ensures a more reliable and unbiased selection of the most appropriate model, avoiding potential misinterpretation that may arise from reliance on a single statistical parameter.

#### 2.6.1. Kinetic models

The adsorption kinetics were systematically analyzed using multiple kinetic models, including the pseudo-first-order model (PFO), pseudo-second-order model (PSO), Avrami kinetic model, Elovich model, and Weber–Morris intraparticle diffusion model. These models were selected to capture different aspects of the adsorption process, including surface reaction kinetics, chemisorption behavior, diffusion mechanisms, and surface heterogeneity.

The corresponding linearized forms of these models were employed to estimate key kinetic parameters, including the rate constants (*k*_1_, *k*_2_, *k*_id_), the Avrami exponent (*n*), the Elovich constants (*α* and *β*), and the equilibrium adsorption capacity (*q*_e_,_cal_). The Weber–Morris model was further used to evaluate the contribution of intraparticle diffusion and to identify potential rate-controlling steps based on multi-linear behavior.

The applicability and predictive performance of each kinetic model were assessed using multiple statistical criteria, including the coefficient of determination (*R*^2^), chi-square (*χ*^2^), root mean square error (RMSE), and Akaike information criterion (AIC). This multi-parameter evaluation provides a robust and unbiased comparison of model performance, enabling reliable identification of the dominant kinetic mechanism governing the adsorption process.

#### 2.6.2. Isotherm models

The equilibrium adsorption behavior was comprehensively analyzed using several isotherm models, including the Langmuir isotherm, Freundlich isotherm, Temkin isotherm, Sips isotherm, Toth isotherm, and Dubinin–Radushkevich isotherm. These models were selected to describe a wide range of adsorption behaviors, from ideal monolayer adsorption on homogeneous surfaces to heterogeneous and energetically non-uniform systems.

Model parameters were estimated using non-linear regression by minimizing the sum of squared errors (SSE) between experimental and predicted values. This approach avoids potential bias associated with linear transformation and ensures more accurate parameter estimation across the entire concentration range.

The calculated parameters include the maximum adsorption capacity (*q*_max_) and Langmuir affinity constant (*b*), Freundlich constants (*K*_*F* and *n*), Temkin constants related to adsorption heat, as well as heterogeneity parameters associated with the Sips and Toth models. In addition, the Dubinin–Radushkevich isotherm was employed to estimate the mean adsorption energy (*E*), providing insight into the adsorption mechanism (physisorption *vs.* chemisorption).

The dimensionless separation factor (*R*_*L*), derived from the Langmuir model, was calculated to evaluate adsorption favorability over the investigated concentration range. The goodness-of-fit and model performance were assessed using multiple statistical criteria, including the coefficient of determination (*R*^2^), chi-square (*χ*^2^), root mean square error (RMSE), and Akaike information criterion (AIC), enabling robust comparison and ranking of competing models.

Nonlinear regression was implemented in *R* using the Levenberg–Marquardt algorithm. This approach enables robust parameter estimation by minimizing the sum of squared residuals between experimental and model-predicted values.

#### 2.6.3. Thermodynamic analysis

Standard Gibbs free energy (Δ*G*°), enthalpy (Δ*H*°), and entropy (Δ*S*°) were determined using the Van't Hoff equation. These parameters were derived from the slope and intercept of the ln *K*_D_*versus* 1/*T* plot, providing insight into the feasibility and energetic nature of the adsorption process.

### 2.7. Artificial neural network (ANN) modeling

#### 2.7.1. Dataset preparation and preprocessing

An artificial neural network (ANN) model was developed to simulate and predict the removal efficiency (% *H*) of methylene blue adsorption onto ZnO-ACRPS. The experimental dataset included five independent input variables: solution pH, contact time (min), adsorbent dosage (g L^−1^), initial MB concentration (mg L^−1^), and temperature (K). The model output was the removal efficiency (%).

The complete dataset consisted of 49 independent adsorption experiments, each representing a unique combination of operational parameters (pH, contact time, adsorbent dosage, initial MB concentration, and temperature). These 49 runs were used as the input–output dataset for ANN modeling. The dataset was randomly divided into training (70%, *n* = 34), validation (15%, *n* = 7), and testing (15%, *n* = 8) subsets to ensure robust model development and unbiased performance evaluation. A fixed random seed was applied to ensure reproducibility of the data partitioning process. Although the dataset size was moderate, model robustness and generalization ability were carefully assessed using repeated 5-fold cross-validation and Leave-One-Out Cross-Validation (LOOCV). These validation strategies minimized overfitting and ensured predictive stability across different data partitions.

#### 2.7.2. Network architecture and hyperparameter optimization

The ANN was implemented as a feedforward multilayer perceptron (MLP) consisting of an input layer with five neurons, one hidden layer, and a single output neuron.

The optimal number of hidden neurons was determined through grid search combined with cross-validation, exploring configurations from 5 to 20 neurons. Model selection was based on minimum validation error and overall generalization performance to avoid overfitting.

The hidden layer employed a hyperbolic tangent sigmoid activation function (tansig), while a linear activation function (purelin) was used in the output layer to allow continuous response prediction.

#### 2.7.3. Model training, cross-validation, and performance evaluation

Network training was carried out using the Levenberg–Marquardt backpropagation algorithm.

Model robustness and predictive stability were further evaluated using repeated 5-fold cross-validation and LOOCV. The final model performance was assessed using the independent test dataset.

Predictive accuracy was quantified using the coefficient of determination (*R*^2^), root mean square error (RMSE), and mean absolute error (MAE):

5 RMSE = sqrt[(1/*N*) × Σ(*y*_pred,*i* − *y*_exp,*i*)^2^]

6 MAE = (1/*N*) × Σ|*y*_pred,*i* − *y*_exp,*i*|

7 *R*^2^ = 1 − [Σ(*y*_exp,*i* − *y*_pred,*i*)^2^/Σ(*y*_exp,*i* − *y*_mean_exp)^2^]

where *y*_exp,*i* and *y*_pred,*i* represent the experimental and predicted values, respectively; *y*_mean_exp is the mean experimental response; and *N* is the total number of observations. All computations were performed in *R* using specialized neural network and statistical packages.

#### 2.7.4. Sensitivity and feature importance analysis

The relative contribution of each input variable (pH, contact time, adsorbent dosage, initial concentration, and temperature) was quantified using Garson's algorithm based on connection weight partitioning. The results were further validated through partial derivative sensitivity analysis.

Relative importance values were normalized to 100% to facilitate comparison of the influence of each predictor on the model output.

### 2.8. Regeneration study

#### 2.8.1. Desorption procedure

After adsorption, saturated ZnO-ACRPS was regenerated using solvent desorption. The spent material was washed with 70% (v/v) ethanol under gentle stirring to disrupt interactions between adsorbed MB molecules and the surface.

The material was then rinsed thoroughly with distilled water to remove residual solvent and remaining species, followed by drying to constant weight before reuse.

#### 2.8.2. Reusability cycles

Reusability was evaluated over three consecutive adsorption–desorption cycles under identical conditions (50 mg L^−1^ MB, pH 7, 0.05 g adsorbent, 25 mL, 25 ± 1 °C). After each cycle, the adsorbent was regenerated with 70% ethanol (60 min stirring), washed, and dried at 80 °C for 12 h.

Structural stability after repeated use was assessed by XRD analysis of the material after the third cycle and compared with fresh ZnO-ACRPS. Regeneration efficiency was calculated based on retained removal performance relative to the first cycle.

### 2.9. Statistical analysis

All experiments were conducted at least in triplicate, and results are presented as mean ± standard deviation (SD).

For ANN modeling, performance metrics (*R*^2^, RMSE, MAE) were calculated using the independent test dataset to ensure objective predictive evaluation. All statistical analyses were performed using R version 4.5.2 (R Foundation for Statistical Computing, Vienna, Austria). Graphical outputs were generated using Origin Pro 2019 and R, with RStudio (Posit, Boston, MA, USA) as the integrated development environment.

## 3. Results and discussion

### 3.1. Structural and surface characterization

#### 3.1.1. Morphology and elemental composition (SEM-EDS, TEM)

**Fig. 2:**
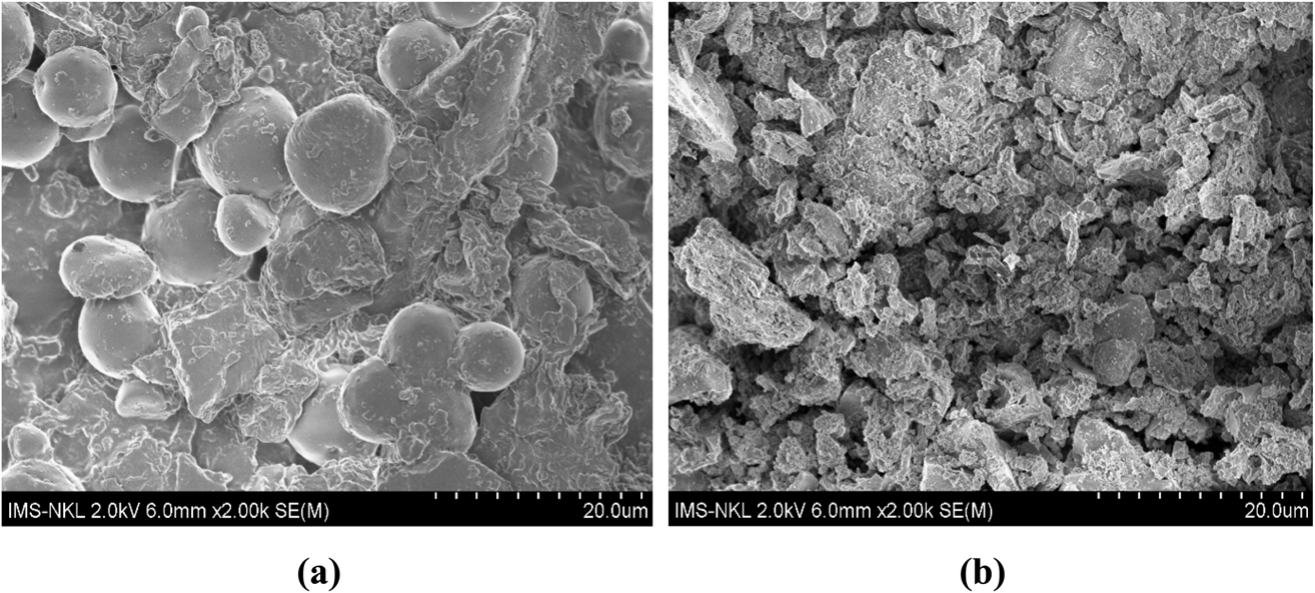
SEM images of ACRPS (a) and ZnO-ACRPS (b).

The surface morphology of ACRPS and ZnO-ACRPS was examined by scanning electron microscopy (SEM), as shown in Fig. 2. The SEM image of ACRPS reveals that the material consists of nearly spherical particles with non-uniform distribution and a tendency to agglomerate into clusters of various sizes (Fig. 2a). The particle surfaces appear relatively rough, with numerous wrinkles and grooves, reflecting the intrinsic carbon structure of biomass-derived activated carbon.

This morphology is formed during carbonization and activation, where the original plant cellular structure undergoes decomposition, shrinkage, and rearrangement under high temperature, leading to the formation of rounded carbon aggregates that minimize surface energy.

Although the observed particles are in the micrometer scale, each particle inherently contains a well-developed microporous network. This internal microporous structure is the primary contributor to the large specific surface area of the material. Such characteristics are consistent with the nature of biomass-derived activated carbon, in which micropores are generated through the removal of volatile components and the restructuring of the carbon framework during activation. Similar morphological features have been widely reported for activated carbons prepared from agricultural residues and lignocellulosic biomass.^40,41^

The SEM image of ZnO-ACRPS (Fig. 2b) shows significant changes in surface morphology compared with pristine ACRPS. On the nearly spherical carbon particles, numerous smaller particles with spherical or spherical shapes are observed, relatively uniformly distributed across the surface and within surface grooves. These particles are attributed to ZnO nanoparticles successfully anchored onto the carbon framework.

The presence of ZnO renders the surface rougher and more porous, while simultaneously introducing new active sites, thereby enhancing interactions with MB molecules and demonstrating superior adsorption potential compared with the original ACRPS. The deposition of ZnO nanoparticles increases surface roughness and introduces additional heterointerfaces.

**Fig. 3:**
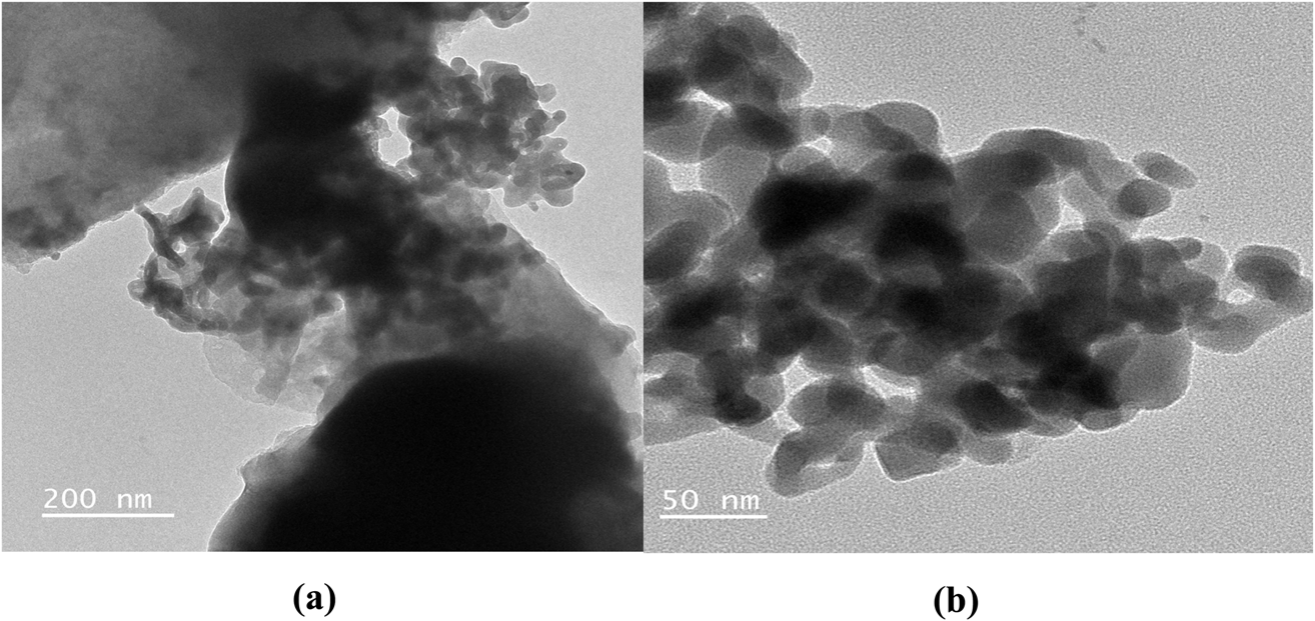
TEM images of ZnO-ACRPS (a) and ZnO (b).

TEM images of ZnO-ACRPS (Fig. 3a) show a heterogeneous carbon matrix with alternating bright and dark regions, reflecting variations in electron density within the carbon network. This feature is consistent with a turbostratic carbon structure commonly observed in low-crystallinity carbon materials.^42^ Such a structure indicates the presence of voids and gaps within the carbon matrix, contributing to a large surface area and a well-developed pore system favorable for adsorption.^9^

TEM images of ZnO-ACRPS (Fig. 3a) show a heterogeneous carbon matrix with alternating bright and dark regions, reflecting variations in electron density within the carbon network. This feature is consistent with a turbostratic carbon structure commonly observed in low-crystallinity carbon materials.^42^ Such a structure indicates the presence of voids and gaps within the carbon matrix, contributing to a large surface area and a well-developed pore system favorable for adsorption.^9^

High-contrast particles are clearly observed on the surface and edges of carbon fragments and are assigned to ZnO nanoparticles. This interpretation is consistent with XRD results confirming the presence of ZnO with a wurtzite structure. The ZnO particles are nanosized, estimated to be in the range of 10–40 nm (Fig. 3b), and are in intimate contact with the carbon matrix, indicating that ACRPS serves as an effective support that limits ZnO agglomeration.

SEM and TEM observations demonstrate that nanoscale ZnO particles are anchored and distributed on the ACRPS surface, while EDX spectra confirm the clear presence of Zn after modification.

**Fig. 4:**
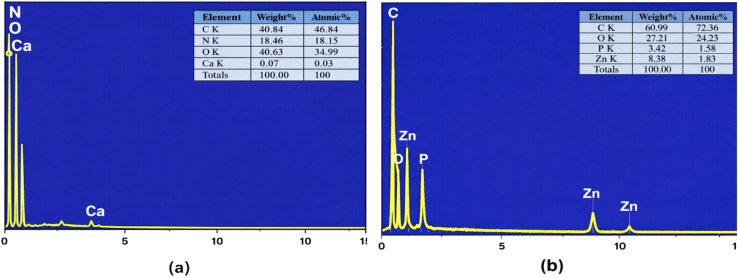
EDS spectra of ACRPS (a) and ZnO-ACRPS (b).

The elemental composition of ZnO-ACRPS and ACRPS was confirmed by EDS analysis (Fig. 4a and b). The EDX spectrum of ACRPS (Fig. 4a) shows that carbon (C) and oxygen (O) are the dominant elements, characteristic of biomass-derived activated carbon, along with a small amount of mineral elements such as Ca, typically present as inorganic ash residues after carbonization.

After modification, the EDX spectrum of ZnO-ACRPS (Fig. 4b) clearly displays characteristic Zn peaks, including Zn–L peaks at low energy (∼1 keV) and Zn–K peaks at higher energy (∼8–10 keV), confirming the successful anchoring of ZnO onto the activated carbon surface.

Simultaneously, the carbon content of ZnO-ACRPS increased (from 40.84% to 60.99%), while the oxygen content decreased (from 40.63% to 27.21%) compared with ACRPS. This variation can be explained by: (i) partial coverage of oxygen-containing functional groups by ZnO particles; (ii) partial decomposition or reduction of oxygen-rich groups during thermal treatment; and (iii) the semi-quantitative nature of EDX analysis, where the presence of heavy Zn alters the relative percentage of lighter elements.^43,44^ The variation in relative elemental percentages should therefore be interpreted cautiously due to the semi-quantitative nature of EDS.

These findings, together with SEM, TEM analysis, demonstrate that ZnO modification significantly alters the elemental composition and surface properties of the material, thereby enhancing its adsorption potential.

#### 3.1.2. Crystalline structure analysis (XRD)

**Fig. 5:**
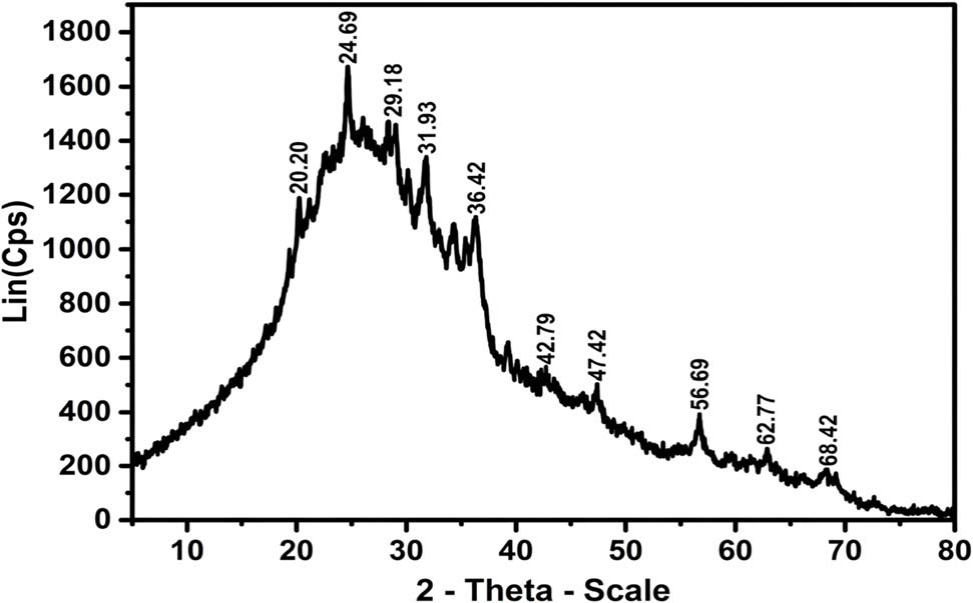
XRD pattern of ZnO-ACRPS.

The XRD pattern of ZnO-ACRPS (Fig. 5) exhibits a broad diffraction peak at 2*θ* ≈ 24.69°, characteristic of the (002) plane of carbon. The calculated interlayer spacing *d*_002_ is approximately 0.36 nm, significantly larger than that of crystalline graphite (0.335 nm), indicating a turbostratic carbon structure with low crystallinity, typical of biomass-derived activated carbons.^45^ In addition to the broad carbon diffraction band, sharp diffraction peaks are observed at 2*θ* ≈ 31.93°, 36.42°, 47.42°, 56.69°, 62.77°, and 68.42°, corresponding to the (100), (101), (102), (110), (103), and (112) planes of wurtzite-structured ZnO, consistent with JCPDS PDF no. 79-0206.^46^ The simultaneous presence of characteristic diffraction peaks of both carbon and ZnO confirms the successful formation and dispersion of ZnO on the ACRPS support.^47^

#### 3.1.3. Surface area and porosity (BET)

**Fig. 6:**
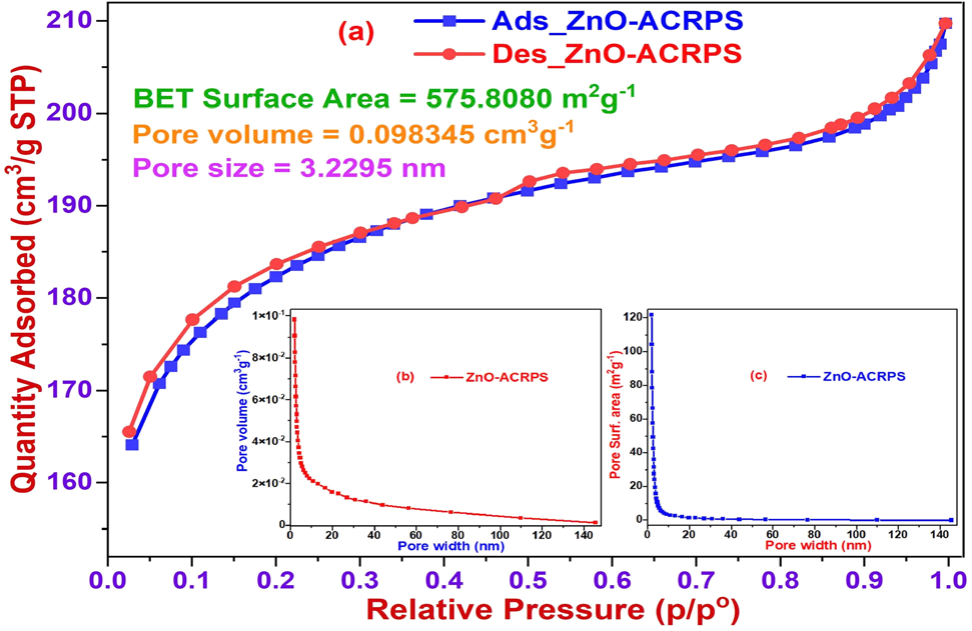
Nitrogen adsorption–desorption isotherm of ZnO-ACRPS.

The N_2_ adsorption–desorption isotherm of ZnO-ACRPS (Fig. 6) shows a sharp increase in adsorption capacity at low relative pressure (*p*/*p*_0_ < 0.1), characteristic of micropore filling. At intermediate to high *p*/*p*_0_ values, nitrogen uptake increases gradually without a distinct capillary condensation step, and a narrow hysteresis loop indicates limited mesopore contribution.

According to IUPAC classification, the isotherm corresponds to a transitional type between I(b) and II, suggesting a heterogeneous pore structure dominated by micropores with the presence of small mesopores.

BET analysis reveals a high specific surface area of 575.81 m^2^ g^−1^, an average pore diameter of 3.22 nm, and a total pore volume of 0.098 cm^3^ g^−1^. These features are favorable for diffusion and adsorption of organic molecules in aqueous environment.^48^

#### 3.1.4. Functional group analysis (FTIR)

**Fig. 7:**
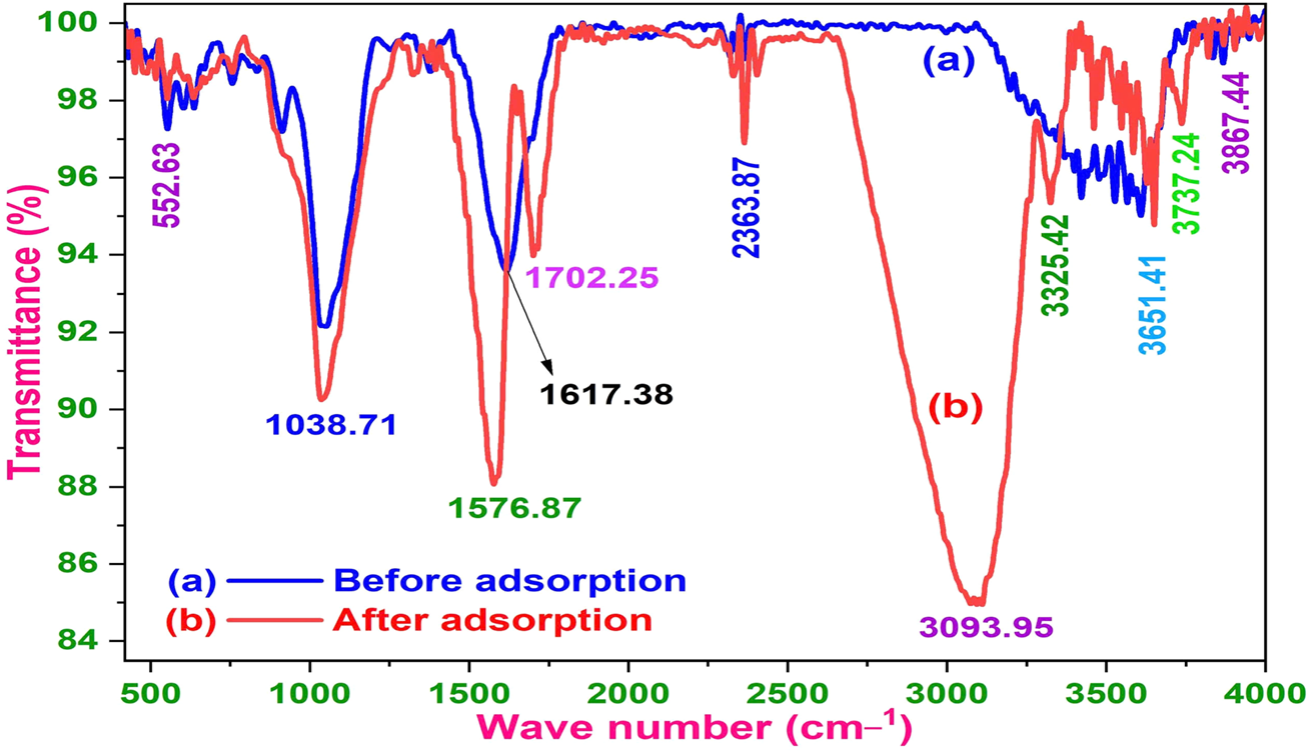
FTIR spectra of ZnO-ACRPS before (a) and after adsorption (b).

FTIR spectra of ZnO-ACRPS before and after MB adsorption are presented in Fig. 7. Before adsorption, a broad band at ∼3608 cm^−1^ corresponds to –OH stretching vibrations from surface hydroxyl groups of carbon and ZnO. This observation is consistent with previous reports on metal oxide-modified carbon materials.^49^ Bands at ∼1698–1617 cm^−1^ are assigned to C=O stretching vibrations (carboxyl/carbonyl groups). The band at ∼1378 cm^−1^ corresponds to symmetric stretching of –COO^−^/–COOH groups, while the band at ∼1254 cm^−1^ is attributed to C–O stretching in phenolic, alcoholic, or ester groups.^50^

A strong band at ∼1050 cm^−1^ is characteristic of C–O–C/C–O vibrations typical of cellulose- and lignin-derived carbon materials.^51,52^ Bands at ∼913–834 cm^−1^ correspond to out-of-plane C–H bending in aromatic structures.^18,53^ The band at ∼553.6 cm^−1^ is attributed to Zn–O vibrations, confirming the presence of ZnO.^54^

After MB adsorption, the –OH band shifts from 3608 to ∼3324 cm^−1^ and becomes broader, suggesting possible hydrogen bonding interactions between the adsorbent surface and MB molecules. The red-shift and band broadening suggest hydrogen bonding interactions with heteroatoms (N, S) and positively charged centers in MB.^55^

A new band at ∼3094 cm^−1^ appears, attributed to aromatic C–H stretching in MB. The absorption band at 1617 cm^−1^, attributed to the stretching vibration of the C=O group (carboxyl/carbonyl), is no longer clearly observed after the adsorption process. The attenuation of this band is most likely due to strong interactions between the surface carbonyl groups and MB molecules, which alter the electronic environment surrounding the C=O bond. This modification reduces the dipole moment variation during vibration, leading to decreased absorption intensity (attenuation) in the FT-IR spectrum, rather than indicating the complete disappearance of the functional group.^55^

The absorption band at ∼1378 cm^−1^, assigned to the symmetric stretching vibration of –COO^−^/–COOH groups, shows reduced intensity, while the band at ∼1254 cm^−1^, attributed to the C–O stretching vibration in phenolic, alcoholic, or ester groups, disappears after adsorption.

New peaks at 1576.87, 1545.05, and 1492 cm^−1^ correspond to aromatic C=C and C–N vibrations of MB, confirming its immobilization on the surface.^49^ The appearance of aromatic ring signals is consistent with possible π–π interactions between MB aromatic rings and graphene-like domains in ACRPS.^56^

The Zn–O band remains after adsorption, indicating that ZnO may provide additional surface sites that contribute to adsorption interactions supporting electrostatic, hydrogen bonding, and π–π interactions rather than simple physical adsorption.^49^

#### 3.1.5. Surface charge properties (pH_pZC_)

**Fig. 8:**
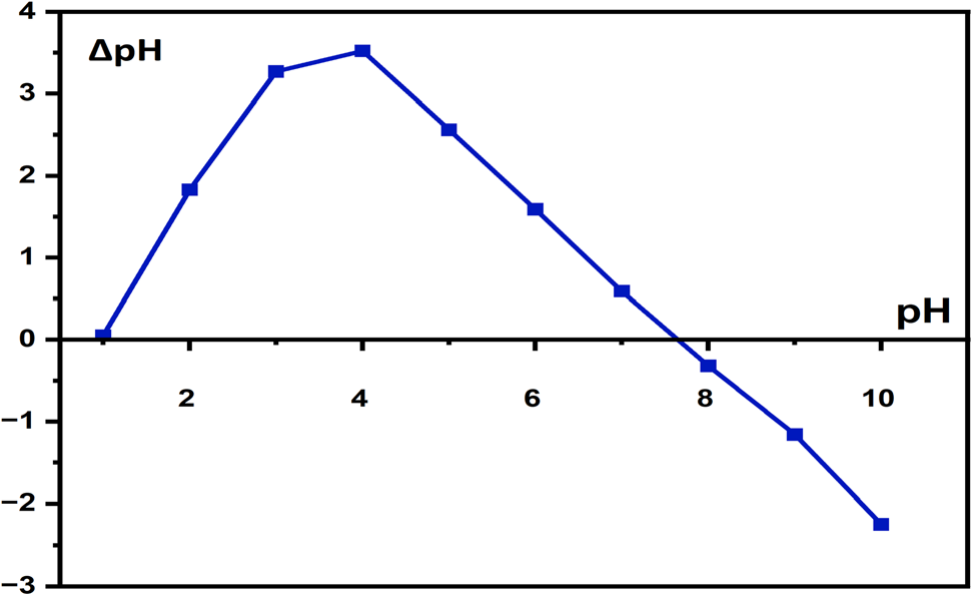
Determination of the pH_pzc_ of ZnO-ACRPS.

The point of zero charge (pH_pzc_) of ZnO-ACRPS was determined to be 7.63 (Fig. 8), indicating that the surface of ZnO-ACRPS is positively charged when the solution pH is lower than this value and negatively charged when the pH exceeds the pH_pzc_. This result is consistent with previous reports on ZnO/activated carbon composites, in which the pH_pzc_ typically ranges from 7.0 to 8.0. For example, Sayed *et al.* (2024)^33^ modified sawdust-derived carbon with ZnO and reported a pH_pzc_ value of 7.5. Marta Mediavilla *et al.*^57^ also determined that the point of zero charge of ZnO- and ZnS-modified banana peel biochar was 7.2. The pH_pzc_ falling within the range of 7–8 can be attributed to the presence of basic Zn–O and Zn–OH groups on the material surface.^58^ Surface Zn–OH groups can undergo protonation–deprotonation equilibria, contributing to amphoteric behavior and shifting the pH_pzc_ toward neutral–alkaline values. Compared with pristine activated carbon (typically with pH_pzc_ < 6),^3^ the increase in pH_pzc_ after ZnO modification indicates that ZnO has been successfully anchored onto the carbon surface and has significantly altered the surface properties of ZnO-ACRPS.

In summary, comprehensive characterization through SEM, TEM, XRD, BET, FTIR, and pH_pzc_ analyses confirms the successful fabrication of the ZnO-ACRPS composite. The results reveal that ZnO nanoparticles (10–40 nm) are uniformly anchored onto the turbostratic carbon matrix, which possesses a well-developed microporous structure and a high specific surface area (575.81 m^2^ g^−1^). ZnO modification not only introduces new active sites but also significantly shifts the point of zero charge (pH_pzc_) to 7.63, thereby altering the surface alkalinity.

Furthermore, FTIR analysis provides compelling evidence of synergistic interactions between the hierarchical hybrid structure and methylene blue (MB) molecules, involving electrostatic attraction, hydrogen bonding, and π–π stacking. These favorable structural and chemical properties highlight the strong potential of ZnO-ACRPS as an efficient adsorbent for the removal of cationic dyes from aqueous environments.

### 3.2. Adsorption performance evaluation

To ensure the repeatability and reliability of the results, each experiment evaluating the adsorption capacity of MB onto ZnO-ACRPS was conducted independently in triplicate under identical conditions. The reported values represent the mean of three measurements.

#### 3.2.1. Effect of pH

**Fig. 9:**
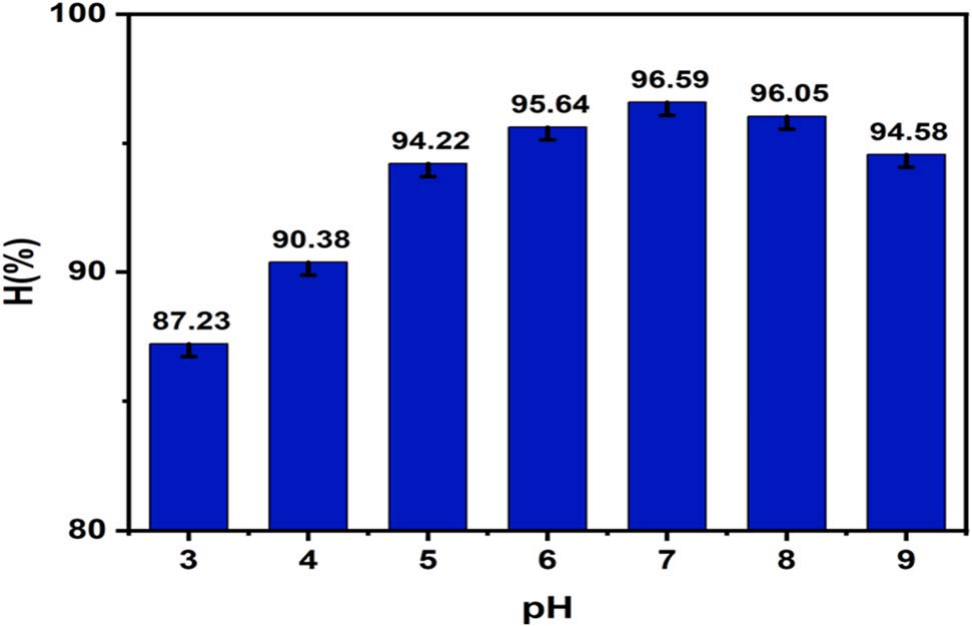
Effect of pH on adsorption efficiency.

Solution pH plays a crucial role in adsorption processes by governing surface charge properties and the speciation of the adsorbate. In particular, the relationship between solution pH and the point of zero charge (pH_pzc_) determines the electrostatic interactions between MB molecules and the ZnO-ACRPS surface. Therefore, evaluating the effect of pH is essential to clarify the dominant adsorption mechanisms in the system. The results of the pH effect are presented in Fig. 9 and S3 (SI).

The adsorption efficiency of MB onto ZnO-ACRPS is strongly influenced by the solution pH and the point of zero charge (pH_pzc_) of the material. With a pH_pzc_ value of 7.63, the surface of ZnO-ACRPS is nearly electrically neutral around neutral pH conditions. As shown in Fig. 9 and S3, the maximum adsorption efficiency was achieved at pH = 7, although this value is slightly lower than the pH_pzc_ of the material. This indicates that MB adsorption is not governed solely by electrostatic interactions but also involves other mechanisms, including π–π interactions between the aromatic rings of MB and the graphitic structure of activated carbon, hydrogen bonding, and surface interactions between MB and the ZnO phase in the composite.^8,59^

When the pH increased to 8 (pH > pH_pzc_), although the negatively charged surface should theoretically favor the adsorption of the cationic MB dye, a slight decrease in adsorption efficiency was observed. This phenomenon may be attributed to the competition of OH^−^ ions with MB for active adsorption sites and possible surface coverage effects, which reduce effective contact between MB and ZnO-ACRPS.^60^ This trend is consistent with previous studies on the adsorption of cationic dyes onto carbon-based materials and AC/metal oxide composites, where the optimal pH is often near neutral and does not necessarily coincide with the pH_pzc_^61,62^.

#### 3.2.2. Adsorption equilibrium time

**Fig. 10:**
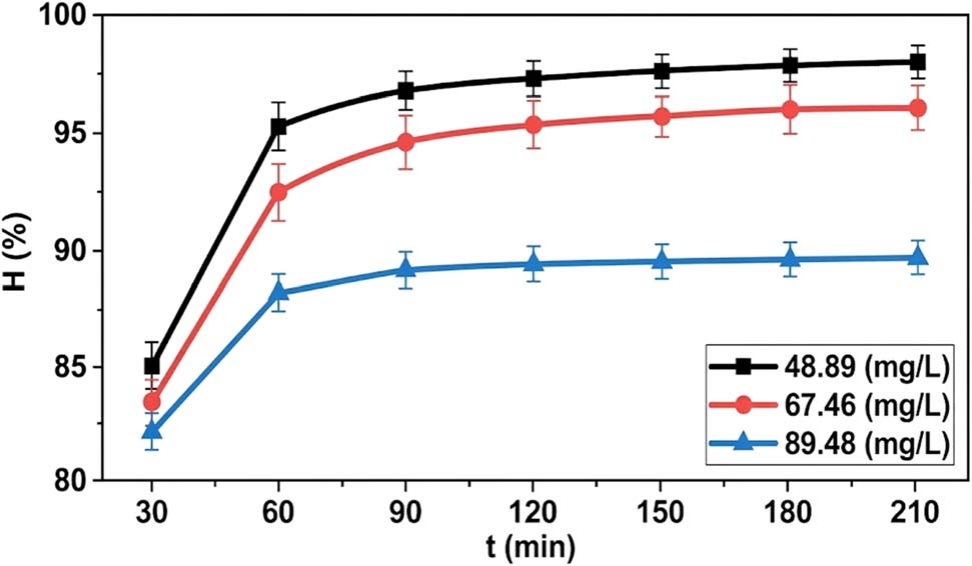
Effect of contact time on adsorption efficiency.

Contact time is a fundamental parameter in adsorption studies, as it reflects the rate of adsorbate uptake and determines the time required to reach equilibrium. Evaluating the time-dependent behavior of MB adsorption provides insight into mass transfer characteristics and surface interaction dynamics of the ZnO-ACRPS system. The results of the time effect are shown in Fig. 10 and S4.

The results in Fig. 10 and S4 (SI) show that MB adsorption onto ZnO-ACRPS increased rapidly during the initial stage (30–120 minutes), followed by a slower increase and gradual approach to equilibrium at longer contact times (≥120 minutes). In the initial stage, the adsorbent surface contains a large number of vacant active sites, and the significant concentration gradient between MB in solution and on the ZnO-ACRPS surface provides a strong diffusion driving force, resulting in a high adsorption rate. As contact time increases, active sites gradually become occupied, reducing the adsorption rate until equilibrium is reached when most adsorption sites are filled. This behavior reflects typical adsorption kinetics and is consistent with previously reported studies on MB adsorption using ZnO/carbon composite materials.^49^ Therefore, an optimal contact time of 120 minutes was selected for MB adsorption onto ZnO-ACRPS.

#### 3.2.3. Effect of adsorbent dosage

**Fig. 11:**
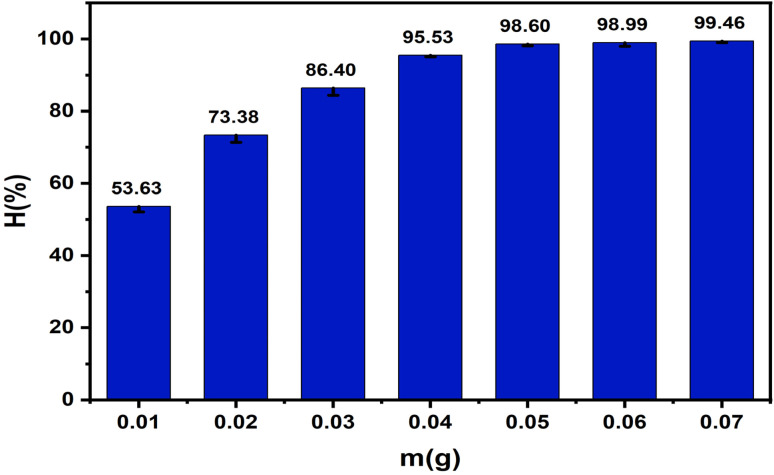
Effect of ZnO-ACRPS dosage on adsorption efficiency.

Adsorbent dosage is a key operational parameter that directly influences the availability of active sites and the overall removal efficiency. Increasing the dosage generally enhances the effective surface area and adsorption capacity, but excessive amounts may lead to diminishing returns due to system saturation. Therefore, evaluating the influence of ZnO-ACRPS dosage is essential to determine the optimal operating conditions. The results presented in Fig. 11 and S5 (SI) show that the adsorption efficiency of MB increased with increasing ZnO-ACRPS dosage. When the adsorbent mass increased from 0.01 to 0.05 g, the adsorption efficiency rose significantly, indicating that the increased number of available active sites on the material surface substantially enhanced MB removal from the solution. However, when the dosage was further increased from 0.05 to 0.125 g, the adsorption efficiency increased only slightly from 98.60% to 99.46%, suggesting that the adsorption system was approaching saturation.

Therefore, the optimal ZnO-ACRPS dosage was selected as 0.05 g for 25 mL of solution, corresponding to 20 g L^−1^. This trend reflects a typical adsorption behavior in which increasing adsorbent dosage enhances the effective surface area and the number of active adsorption sites, thereby improving MB removal efficiency, consistent with previous studies on similar carbon-based and composite materials.^63^ Once the ZnO-ACRPS dosage reaches a certain level, most MB molecules in the solution have already been captured on the material surface; consequently, additional vacant adsorption sites no longer contribute significantly to further improvement in removal efficiency. As a result, the adsorption efficiency remains nearly constant with further increases in adsorbent mass. This phenomenon indicates that adsorption efficiency increases with dosage at the initial stage but subsequently approaches a plateau due to saturation of the effectively active adsorption sites.^64^

#### 3.2.4. Effect of shaking speed

**Fig. 12:**
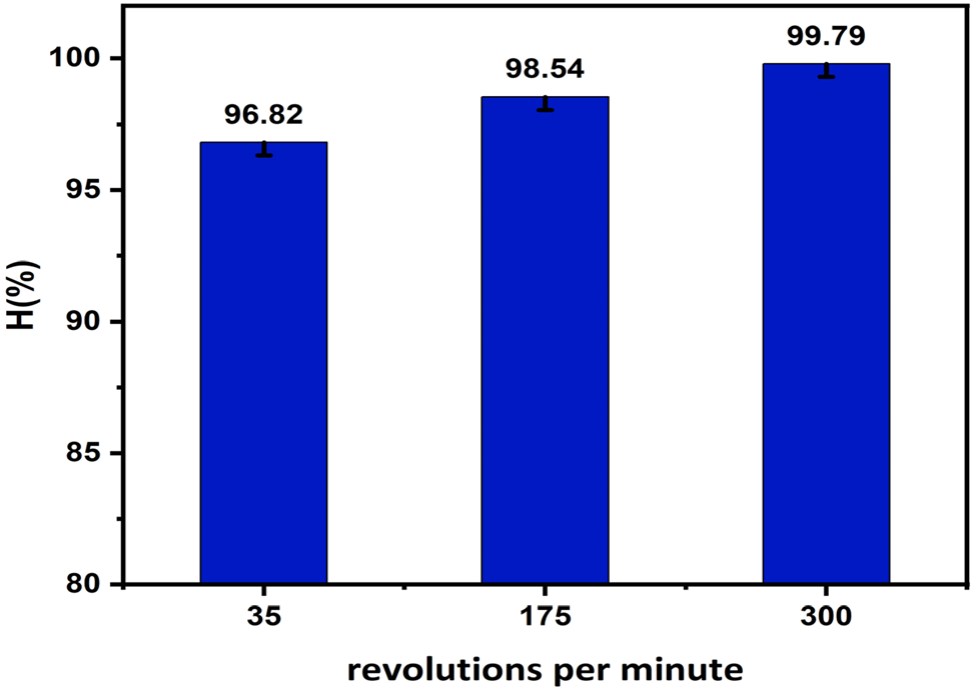
Effect of shaking speed on adsorption efficiency.

Agitation speed affects adsorption performance by influencing external mass transfer resistance and the contact efficiency between adsorbate molecules and the adsorbent surface. Increasing shaking speed generally enhances mixing and reduces the boundary layer thickness surrounding the adsorbent particles. Therefore, evaluating the effect of shaking speed is important to clarify diffusion limitations and determine the optimal operational condition. The effect of shaking speed was investigated, and the results are presented in Fig. 12 and S6 (SI).

The results shown in Fig. 12 indicate that shaking speed influences MB adsorption efficiency on ZnO-ACRPS through mass transfer mechanisms between the solution and the material surface. When the shaking speed increased from 35 to 175 rpm, the adsorption efficiency improved from 96.68% to 98.85%, demonstrating that enhanced agitation reduces external diffusion resistance and improves contact between MB molecules and active adsorption sites on ZnO-ACRPS.

However, when the shaking speed was further increased to 300 rpm, the adsorption efficiency increased only slightly to 99.06%, indicating that the adsorption system was approaching equilibrium and was no longer significantly controlled by external mass transfer. At this stage, the adsorption process is mainly limited by the number of available adsorption sites and intraparticle diffusion mechanisms, which are less affected by agitation speed.

Therefore, a shaking speed of 175 rpm was selected as the optimal condition for subsequent experiments to ensure high adsorption efficiency while minimizing energy consumption and unnecessary mechanical stress on the material. This trend is consistent with previously reported studies, where adsorption efficiency typically increases rapidly at low agitation speeds and approaches a maximum value beyond a certain mixing threshold.^8,65^

#### 3.2.5. Effect of temperature

**Fig. 13:**
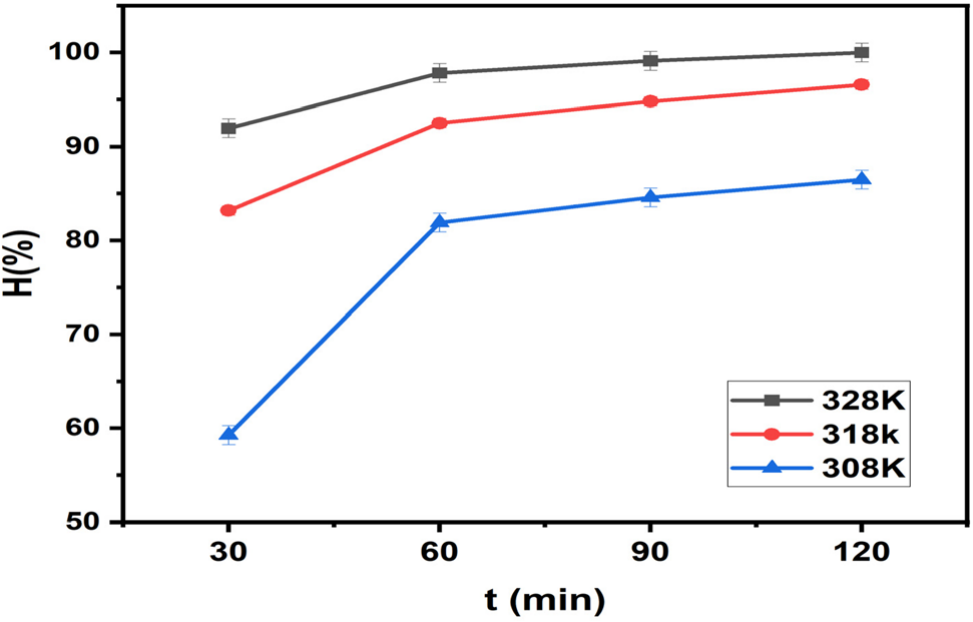
Effect of temperature on adsorption efficiency.

Temperature is a key parameter influencing adsorption kinetics and thermodynamic behavior. Changes in temperature affect molecular diffusion, solution viscosity, and the interaction strength between MB molecules and active sites on ZnO-ACRPS. Therefore, investigating temperature dependence provides insight into the adsorption mechanism of the system. The results are presented in Fig. 13 and S7 (SI).

The results shown in Fig. 13 and S7 (SI) indicate that temperature exerts a noticeable influence on the adsorption efficiency of MB onto ZnO-ACRPS. The increase in adsorption efficiency with rising temperature can be attributed to the higher thermal energy, which enhances the kinetic energy of MB molecules, thereby promoting their diffusion from the bulk solution to the ZnO-ACRPS surface and facilitating their penetration into the pore system of the material. In addition, elevated temperature reduces the viscosity of the solution, improving effective contact between MB molecules and active adsorption sites on the material surface.^3,4^

Similar trends have been reported in numerous studies on dye adsorption using carbon-based and metal oxide composite materials, where increasing temperature enhances the diffusivity of the adsorbate and strengthens both the extent and duration of interaction between adsorbate molecules and the adsorbent surface.^66^

The observed increase in MB adsorption efficiency with temperature suggests that the adsorption process on ZnO-ACRPS is endothermic in nature. Thermal energy supplied from the surroundings helps overcome energy barriers associated with intraparticle diffusion and the formation of interactions between MB molecules and adsorption sites on the material surface. However, the magnitude of the efficiency increase is relatively modest, indicating that the process is primarily limited by the number of available adsorption sites and that physisorption plays a dominant role compared with chemisorption. Once the temperature reaches a certain threshold, the adsorption system approaches equilibrium, and further temperature increases do not significantly improve adsorption efficiency due to gradual saturation of effective adsorption sites.^8^

#### 3.2.6. Effect of initial MB concentration

**Fig. 14:**
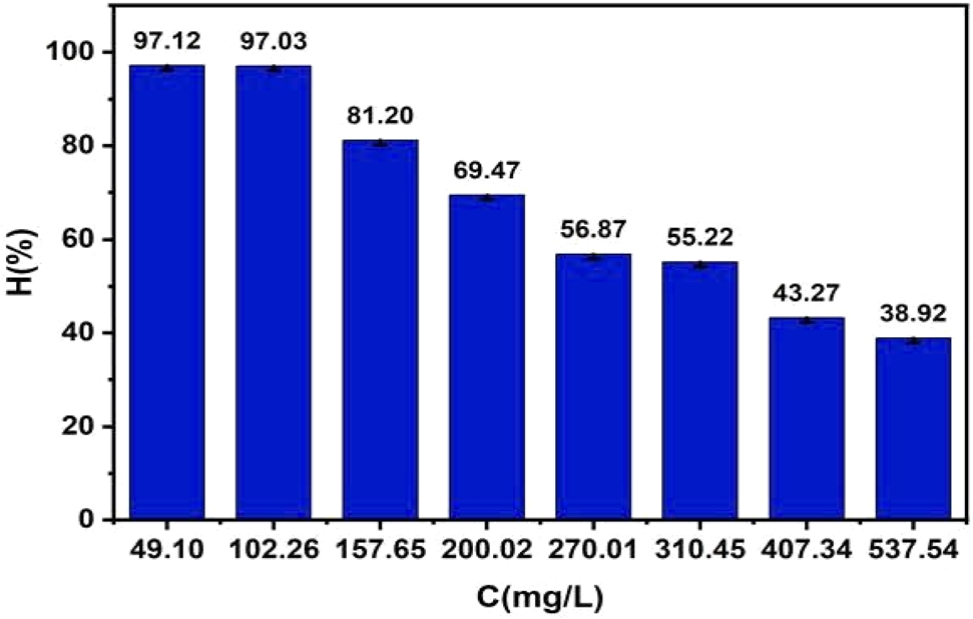
Effect of initial concentration on adsorption efficiency.

Initial adsorbate concentration is a critical factor influencing adsorption performance, as it determines the driving force for mass transfer and the degree of competition for active sites. Variations in concentration affect the interaction dynamics between MB molecules and the ZnO-ACRPS surface, particularly under conditions of site limitation. Therefore, investigating the effect of initial MB concentration is essential to understand adsorption capacity and system behavior under different loading conditions. The results are presented in Fig. 14 and S8 (SI). The results shown in Fig. 14 and S8 (SI) demonstrate that the adsorption efficiency of MB onto ZnO-ACRPS gradually decreases as the initial pollutant concentration increases from 49.10 to 537.54 mg L^−1^. At low initial concentrations, the number of available adsorption sites on the ZnO-ACRPS surface greatly exceeds the number of MB molecules in solution, facilitating effective binding and resulting in high adsorption efficiency.

As the initial concentration increases, active adsorption sites become progressively occupied and approach saturation. Meanwhile, competition among MB molecules for the available adsorption sites intensifies, leading to a reduction in overall adsorption efficiency. This phenomenon reflects the typical behavior of adsorption systems with a finite number of active sites and is consistent with the Langmuir and Freundlich isotherm models, as reported by previous studies.^67,68^

### 3.3. Adsorption isotherms

**Fig. 15:**
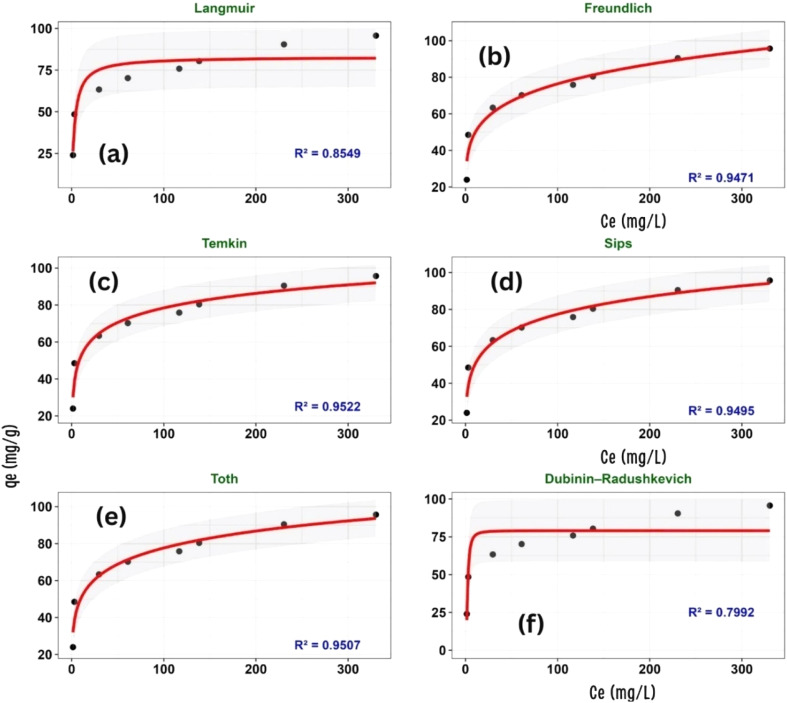
Nonlinear fitting of adsorption isotherm models: (a) Langmuir, (b) Freundlich, (c) Temkin, (d) Sips, (e) Toth, and (f) Dubinin–Radushkevich. Symbols represent experimental data and solid lines represent model fits.

The relationship between equilibrium adsorption capacity (*q*_e_) and equilibrium concentration (*C*_e_), together with the nonlinear isotherm models (Langmuir, Freundlich, Temkin, Dubinin–Radushkevich, Sips, and Toth), is illustrated in Fig. 15 and Table 1. The fitted parameters of these nonlinear isotherm models are presented in Table 2.

**Table 1:** Non-linear isotherm parameters for methylene blue (MB) adsorption onto ZnO-ACRPS, obtained from Langmuir, Freundlich, Temkin, Sips, Toth, and Dubinin–Radushkevich models

Parameter	Langmuir	Freundlich	Temkin	Sips	Toth	Dubinin–Radushkevich
*q*_max_ (mg g^−1^)	82.95			222.75	333.58	
*K*_L_ (L mg^−1^)	0.326					
*R*_L_	0.058					
*K*_F_		31.93				
*n*		5.28				
*A* (L g^−1^)			9.97			
*B* (J mol^−1^)			11.36			
*K*_S_ (L mg^−1^)					3841.24	
*K*_T_ (L mg^−1^)					0.125	
*t*				0.266		
*K*_Sips_ (L mg^−1^)				9.33 × 10^−4^		
*q*_m_ (mg g^−1^)						79.05
*β* (mol^2^ kJ^−2^)						7.88 × 10^−7^

**Table 2:** Statistical evaluation and ranking of isotherm models^a^

Model	*χ* ^2^	*R* ^2^	RMSE (mg g^−1^)	AIC	ΔAIC	Rank
Temkin	**4.31**	**0.9522**	**4.78**	**29.03**	**0.00**	**1**
Freundlich	5.35	0.9471	5.03	29.85	0.82	2
Toth	4.88	0.9507	4.86	31.28	2.25	3
Sips	5.06	0.9495	4.92	31.48	2.45	4
Langmuir	7.72	0.8549	8.33	37.92	8.89	5
Dubinin–Radushkevich	10.48	0.7992	9.80	40.52	11.49	6

a Note: *R*^2^: coefficient of determination; RMSE: root mean square error; AIC: Akaike information criterion; ΔAIC: difference in AIC relative to the best-fitting model; *χ*^2^: chi-square statistic representing the goodness-of-fit of the isotherm model. The model with ΔAIC = 0 is considered the best-fitting model.

The reported parameters represent adsorption capacity (*q*_max_), surface heterogeneity indices, adsorption affinity constants, and energy-related coefficients, providing comprehensive insight into the adsorption mechanism and performance of the developed material.

The isotherm profiles in Fig. 15, together with the statistical metrics in Tables 1 and 2, point to the Temkin model as the closest representation of the experimental data (ΔAIC = 0, *R*^2^ = 0.9522). The better performance of the Temkin model suggests that the adsorption process is associated with a progressive decrease in adsorption energy as surface coverage increases, a feature commonly associated with heterogeneous systems.^69^

In contrast to the Langmuir assumption of a uniform surface with identical binding energies, the superior agreement of the Temkin model implies a distribution of energetically distinct adsorption sites. The ZnO-ACRPS surface accommodates such heterogeneity. Electron microscopy (Fig. 2 and 3) reveals ZnO nanoparticles dispersed over a turbostratic carbon matrix, creating a range of active sites rather than a single uniform environment. High-energy sites are occupied first, followed by progressively lower-energy sites, consistent with the Temkin framework.^69^ The Temkin constants (*A* = 9.97 L g^−1^ and *B* = 11.36 J mol^−1^) are consistent with moderate interactions between adsorbate and adsorbent, although these parameters alone do not allow definitive identification of the adsorption mechanism. Such hybrid behavior frequently appears in metal oxide–carbon composites, where multiple interaction pathways operate simultaneously.^70^ The Freundlich constant (*n* = 5.28) exceeds unity by a wide margin, indicating favorable adsorption and suggesting the presence of heterogeneous surface sites. Elevated *n* values are typically linked to pronounced surface heterogeneity and a tendency toward multilayer adsorption.^70^

The Langmuir model deviates more strongly from the data (ΔAIC ≈ 8.89), indicating that the assumption of a homogeneous surface with identical adsorption sites is not fully applicable to the present system. A similar mismatch appears for the Dubinin–Radushkevich (D–R) model, which yields the lowest correlation (*R*^2^ = 0.799). This result implies that pore-filling is unlikely to be the dominant adsorption mechanism, although contributions from porous structure cannot be entirely excluded. This suggests that surface interactions may play an important role under the studied conditions. However, contributions from pore structure cannot be excluded.

The small ΔAIC differences (<2) among the Temkin, Freundlich, and Toth models indicate that these models provide statistically comparable fits to the experimental data. This suggests that multiple isotherm models can adequately describe the adsorption system, reflecting the inherent complexity of the adsorption process that cannot be fully captured by a single theoretical model. Overall, the isotherm analysis indicates that MB adsorption on ZnO-ACRPS is better described by models that account for surface heterogeneity. The results indicate that adsorption occurs on heterogeneous sites with varying affinities, leading to a deviation from the ideal monolayer adsorption behavior. From the Langmuir model, the maximum adsorption capacity was estimated as *q*_max_ = 82.95 mg g^−1^.

Although the Temkin model provides the best statistical fit, it primarily describes the adsorption mechanism rather than adsorption capacity. The Langmuir model, despite its lower fitting performance, remains important for estimating the theoretical maximum adsorption capacity (*q*_max_), which serves as a standard metric for comparison with other adsorbents. In this study, the maximum adsorption capacity was determined to be 82.95 mg g^−1^.

The combined use of Temkin (mechanistic insight) and Langmuir (capacity evaluation) offers a balanced interpretation of the adsorption process and better reflects the complexity of heterogeneous adsorption systems (Table 3).

**Table 3:** Maximum adsorption capacity (*q*_max_) of ZnO-ACRPS and selected ZnO-modified adsorbents for MB removal

No.	Absorbent material	Adsorption capacity (mg g^−1^)	References
1	Multifunctional grafted polyacrylic acid composite (carboxymethyl chitosan/alginate) (CMCH/ALG)-g-PAA with ZnO-modified graphitic carbon nitride (ZnO-g-C_3_N_4_)	24.30	71
2	ZnO-Zeolite composite	41.32	72
3	H_3_PO_4_-activated hazelnut shell activated carbon modified with ZnO	270.70	73
4	ZnO/CS/CMC composite	4.66	74
5	ZnO-modified banana peel biochar	118.8	75
6	AC-ZnO-NH_3_ composite	106.38	49
7	ZnO-modified pineapple peel waste biomass (ZnONPs/PPWB)	63.43	76
8	ZnO/porous biochar nanocomposite	826.44	62
9	**ZnO-ACRPS**	**82.95**	**This study**

A comparative analysis of MB adsorption capacities for various ZnO-modified materials (Table 1) reveals that ZnO-ACRPS exhibits a moderate adsorption capacity (82.95 mg g^−1^), falling within the range reported for similar systems, although lower than that of certain high-efficiency ZnO-based adsorbents. It is important to note that direct comparisons should be approached with caution, as adsorption performance is highly dependent on experimental parameters such as initial concentration, solution pH, temperature, and adsorbent dosage, which can significantly influence the reported capacities. The variability in reported adsorption capacities reflects differences in material structure, surface chemistry, and experimental conditions rather than intrinsic performance alone.

The dimensionless separation factor (*R*_L_), derived from the Langmuir constant (*K*_L_ = 0.326 L mg^−1^), was calculated as 0.058 (Table 1) at an initial concentration of 49.47 mg L^−1^.The *R*_L_ value (0 < *R*_L_ < 1) indicates favorable adsorption behavior under the studied conditions.

### 3.4. Adsorption kinetics

To investigate the adsorption behavior of MB on the ZnO-ACRPS material, five kinetic models were employed to interpret the experimental data, including pseudo-first-order (PFO), pseudo-second-order (PSO), Elovich, Weber–Morris, and Avrami models. The analysis was conducted using nonlinear forms, which avoid distortions associated with linearization procedures. Such transformations often alter the error structure and may bias the estimation of kinetic parameters. Direct fitting of the original model equations to the experimental data provides a more reliable basis for mechanistic interpretation.

**Fig. 16:**
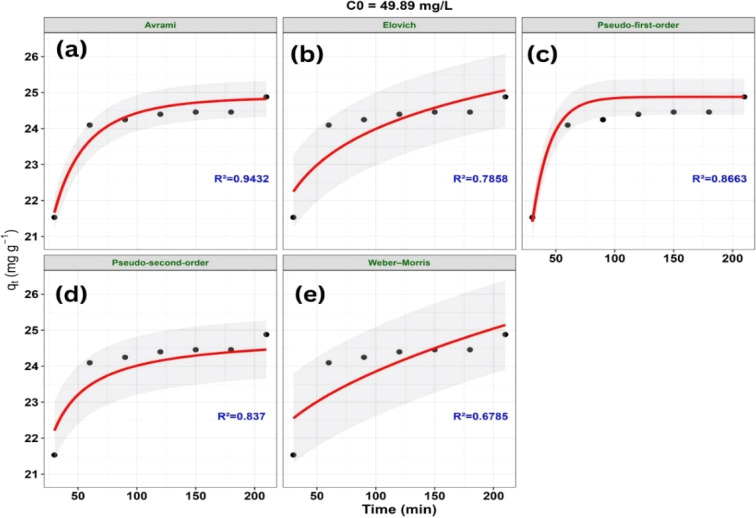
Nonlinear kinetic modeling of methylene blue (MB) adsorption onto ZnO-ACRPS at *C*_0_ = 49.89 mg L^−1^: (a) Avrami, (b) Elovich, (c) pseudo-first-order (PFO), (d) pseudo-second-order (PSO), and (e) Weber–Morris models. Symbols represent experimental data and solid lines represent model fits (shaded areas indicate 95% confidence intervals).

**Fig. 17:**
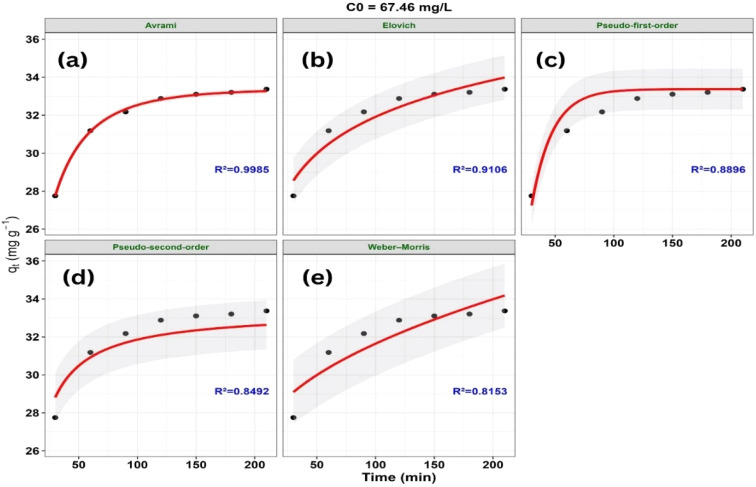
Non-linear kinetic modeling of methylene blue (MB) adsorption onto ZnO-ACRPS at *C*_0_ = 67.46 mg L^−1^: (a) Avrami, (b) Elovich, (c) pseudo-first-order (PFO), (d) pseudo-second-order (PSO), and (e) Weber–Morris models. Symbols represent experimental data and solid lines represent model fits (shaded areas indicate 95% confidence intervals).

**Fig. 18:**
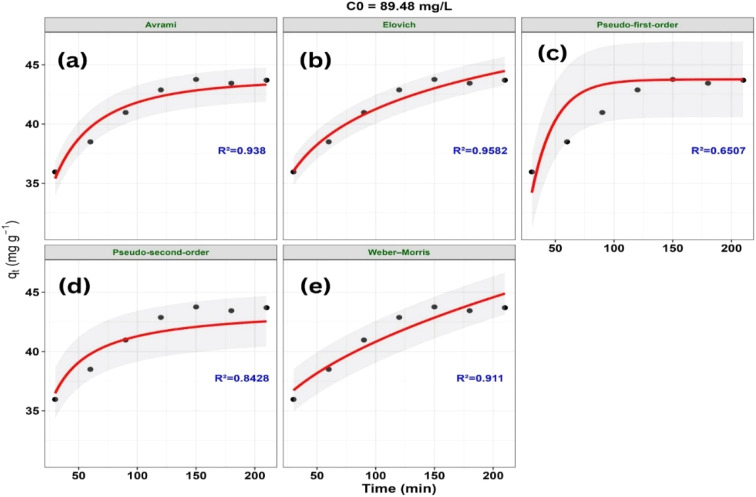
Non-linear kinetic modeling of methylene blue (MB) adsorption onto ZnO-ACRPS at *C*_0_ = 67.46 mg L^−1^: (a) Avrami, (b) Elovich, (c) pseudo-first-order (PFO), (d) pseudo-second-order (PSO), and (e) Weber–Morris models. Symbols represent experimental data and solid lines represent model fits (shaded areas indicate 95% confidence intervals).

The variation of *q*_*t*_ with contact time (*t*) according to the nonlinear kinetic models at different initial concentrations is presented in Fig. 16–18.

A statistical comparison of the kinetic models based on goodness-of-fit criteria (*R*^2^, RMSE, *χ*^2^, AIC, and ΔAIC) at different initial concentrations (*C*_0_) is presented in Table 4.

**Table 4:** Statistical comparison of kinetic models based on goodness-of-fit parameters (*R*^2^, RMSE, *χ*^2^, AIC, and ΔAIC) at different initial concentrations (*C*_0_)^a^

*C*_0_ (mg L^−1^)	Model	*R* ^2^	RMSE	*χ* ^2^	AIC	ΔAIC
**49.89**	**Avrami**	**0.9432**	**0.2468**	**0.0177**	**−15.587**	**0**
	Pseudo-first-order	0.8663	0.3788	0.0406	−11.592	3.995
	Pseudo-second-order	0.8370	0.4181	0.0527	−10.208	5.379
	Elovich	0.7858	0.4793	0.0694	−6.296	9.291
	Weber–Morris	0.6785	0.5873	0.1043	−3.452	12.136
67.46	**Avrami**	**0.9985**	**0.0725**	**0.0011**	**−32.744**	**0**
	Pseudo-first-order	0.8896	0.6160	0.0827	−4.783	27.962
	Elovich	0.9106	0.5544	0.0696	−4.259	28.485
	Pseudo-second-order	0.8492	0.7199	0.1166	−2.601	30.144
	Weber–Morris	0.8153	0.7969	0.1444	0.822	33.567
89.48	**Elovich**	**0.9582**	**0.5748**	**0.0544**	**−3.753**	**0**
	Avrami	0.9380	0.7003	0.0846	−0.987	2.766
	Weber–Morris	0.9110	0.8389	0.1173	1.541	5.294
	Pseudo-second-order	0.8428	1.1152	0.2099	3.526	7.279
	Pseudo-first-order	0.6507	1.6622	0.4745	9.114	12.867

a Note: the model with ΔAIC = 0 is considered the best-fitting model.

The adsorption kinetics of MB on ZnO-ACRPS were examined by comparing experimental data at different initial concentrations with several nonlinear kinetic models, including pseudo-first-order (PFO), pseudo-second-order (PSO), Elovich, Weber–Morris (intraparticle diffusion), and Avrami (Fig. 16–18 and Table 4). Based on goodness-of-fit criteria (*R*^2^, RMSE, *χ*^2^, AIC, and ΔAIC), the Avrami model best described the adsorption kinetics at 49.89 and 67.46 mg L^−1^, with ΔAIC = 0. This result suggests that the adsorption kinetics cannot be adequately described by simple kinetic models and may involve complex adsorption behavior. Similar behavior has been reported in systems where PFO and PSO models do not adequately represent the kinetic profile.^77^ The large ΔAIC differences observed for PFO and PSO models indicate significantly poorer fits compared to the Avrami model.

At the highest concentration (89.48 mg L^−1^), the Elovich model provided the best fit, with ΔAIC = 0. The Elovich model is often associated with adsorption on heterogeneous surfaces and decreasing adsorption rates over time; however, it remains an empirical model and does not provide direct mechanistic evidence. Therefore, this behavior should be interpreted as a descriptive representation of the adsorption process rather than a definitive mechanistic explanation.

The PFO and PSO models show relatively poorer agreement with the experimental data across most concentrations, indicating that simple kinetic models are insufficient to adequately describe the adsorption behavior. The Weber–Morris model does not adequately represent the full adsorption profile, suggesting that intraparticle diffusion may not be the sole rate-controlling step. Therefore, the adsorption process is likely influenced by multiple mass transfer steps; however, these contributions cannot be quantitatively distinguished based solely on the present kinetic models.^77^

Variation in model performance suggests different models describe adsorption under varying conditions, without indicating a definitive mechanism; overall, no single model adequately captures the process.

### 3.5. Thermodynamic behavior

#### 3.5.1. Activation energy

**Fig. 19:**
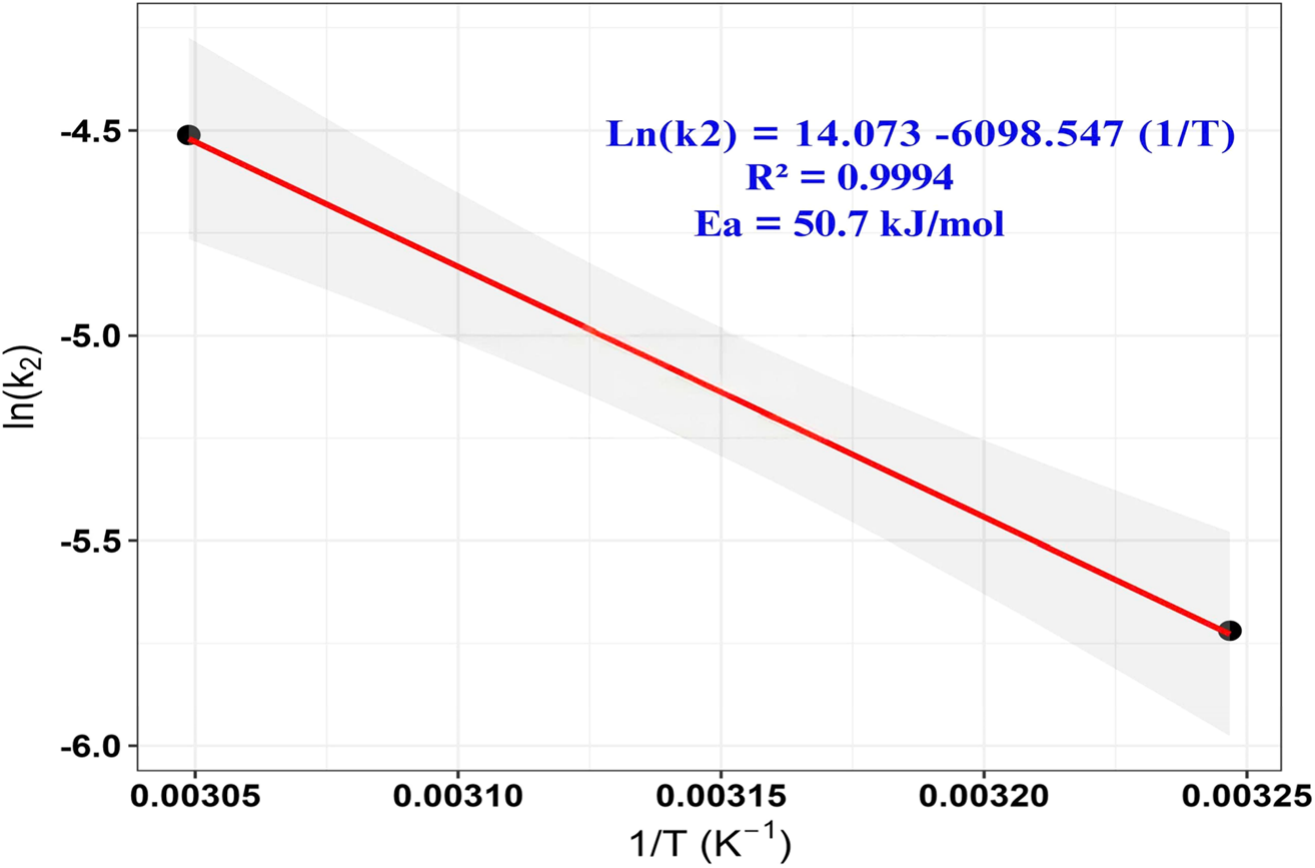
Arrhenius plot of ln(*k*_2_) *versus* reciprocal temperature (1/*T*) for MB adsorption on ZnO-ACRPS. The linear fit (*R*^2^ = 0.9994) yields an activation energy (*E*_a_) of 50.7 kJ mol^−1^.

Arrhenius plot of ln(*k*_2_) *versus* reciprocal temperature (1/*T*) for the adsorption process. The linear relationship indicates that the kinetics follow the Arrhenius equation. The high coefficient of determination (*R*^2^ = 0.9994) suggests an excellent fit, confirming the reliability of the kinetic model. The activation energy (*E*_a_) for MB adsorption on ZnO-ACRPS was determined to be approximately 50.7 kJ mol^−1^ from the Arrhenius plot (Fig. 19).

In adsorption systems, lower *E*_a_ values (typically < 40 kJ mol^−1^) are often associated with physisorption governed by weak intermolecular interactions, whereas higher values may indicate stronger interactions. However, the range of ∼40–80 kJ mol^−1^ represents an overlap region where both physical and chemical interactions may coexist, making mechanistic interpretation non-trivial.^8,9^

Therefore, the obtained *E*_a_ suggests the involvement of moderately strong interactions rather than purely weak physical adsorption. Nevertheless, activation energy alone cannot reliably distinguish between physisorption and chemisorption mechanisms, as adsorption processes often involve multiple concurrent interactions and kinetic contributions.^8,78^

The high linearity of the Arrhenius plot indicates that the temperature dependence of the kinetic constant is well described by the Arrhenius equation. However, a high coefficient of determination reflects only the goodness of fit and does not imply mechanistic uniqueness or confirm a specific adsorption pathway.^9^

#### 3.5.2. Adsorption thermodynamics

**Fig. 20:**
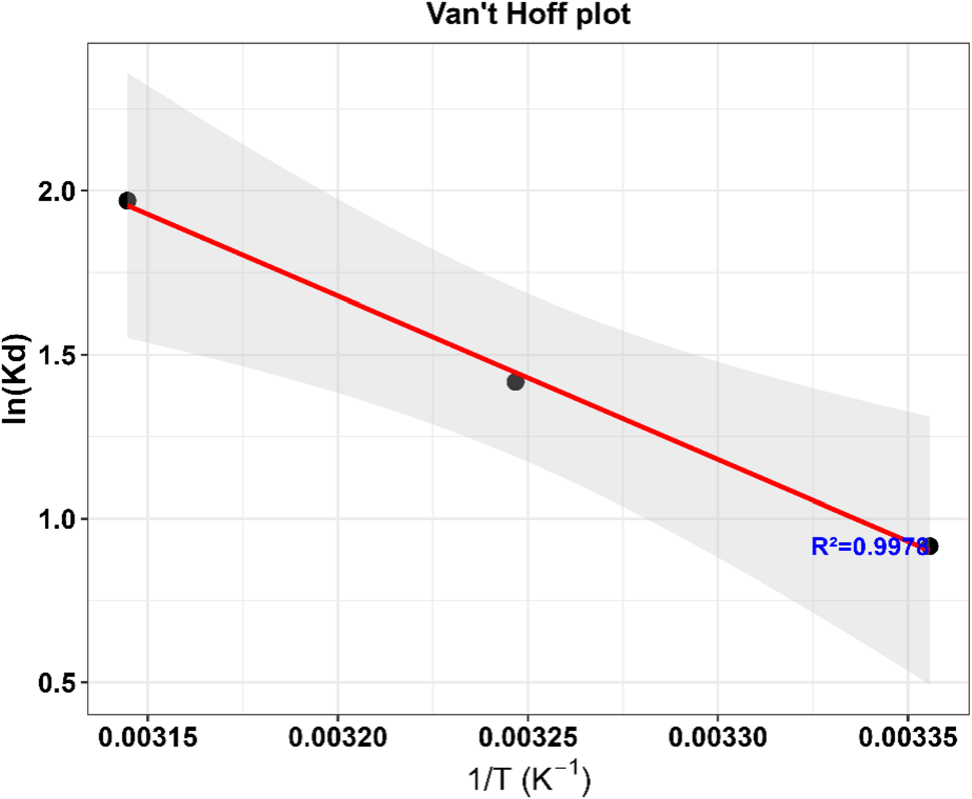
Van't Hoff plot of ln(*K*_d_) *versus* 1/*T* for the adsorption process. Symbols represent experimental data and the solid line denotes the linear fit, from which Δ*H*° and Δ*S*° were obtained.

Thermodynamic parameters for MB adsorption on ZnO-ACRPS were evaluated at 298–318 K and are summarized in Table 5 and Fig. 20. The negative values of Δ*G*° (−2.27 to −5.21 kJ mol^−1^) indicate that the adsorption process is spontaneous, consistent with the fundamental thermodynamic criterion for feasibility.

**Table 5:** Thermodynamic parameters of the MB adsorption process^a^

*T* (K)	Δ*G*^0^ (kJ mol^−1^)	Δ*H*^0^ (kJ mol^−1^)	Δ*S*° (J mol^−1^ K^−1^)
298	−2.270	41.45	146.60
308	−3.629		
318	−5.207		

a Note: thermodynamic parameters including standard Gibbs free energy change (Δ*G*°), enthalpy change (Δ*H*°), and entropy change (Δ*S*°) for the adsorption process at different temperatures.

Moreover, the relatively small magnitude of Δ*G*° suggests that the adsorption is governed predominantly by weak interactions and remains readily reversible, which is commonly observed in dye adsorption systems involving electrostatic attraction and van der Waals interactions.^5^

The positive enthalpy change (Δ*H*° = 41.45 kJ mol^−1^) confirms that the adsorption process is endothermic. This value lies within an intermediate range, indicating that the adsorption is not purely physical but may involve moderately strong interactions such as hydrogen bonding or surface complexation. However, Δ*H*° alone cannot provide a definitive distinction between physisorption and chemisorption mechanisms, as both types of interactions may coexist in heterogeneous adsorption systems.^3^

The positive entropy change (Δ*S*° = 146.60 J mol^−1^ K^−1^) reflects an increase in disorder at the solid–liquid interface during adsorption. This behavior is typically attributed to the displacement of structured water molecules and the reorganization of solvation layers surrounding the adsorbate and adsorbent surface, leading to increased degrees of freedom in the system.

The equilibrium constant (*K*_d_) was approximated from adsorption data (*q*_e_/*C*_e_); however, it should be noted that this definition represents an apparent distribution coefficient rather than a strictly thermodynamic equilibrium constant. Consequently, the derived thermodynamic parameters should be interpreted as semi-quantitative descriptors rather than absolute thermodynamic quantities.^3,6^

Overall, the thermodynamic analysis indicates that MB adsorption on ZnO–ACRPS is spontaneous, endothermic, and entropy-driven. The adsorption process likely involves a combination of weak and moderately strong interactions, including electrostatic attraction, hydrogen bonding, and surface interactions. However, the exact adsorption mechanism cannot be conclusively determined based solely on thermodynamic parameters and should be interpreted in conjunction with kinetic and structural analyses.^3,6^

### 3.6. ANN predictive performance

To ensure reliability and optimal generalization, the experimental dataset was divided into training, validation, and testing subsets at a ratio of 70 : 15 : 15. A 5-fold cross-validation (CV) procedure was applied throughout the training process to minimize bias caused by data partitioning and to confirm model stability prior to detailed performance evaluation. Given the relatively small dataset (*n* = 49), the combined use of cross-validation and residual diagnostics ensures robust generalization within the investigated parameter space.

To further evaluate the predictive capability and robustness of the developed ANN model, the MSE values obtained from the 5-fold CV procedure were analyzed in detail. The corresponding mean squared error (MSE) values for training and validation datasets are summarized in Table S1 and S2 (in SI), while the simultaneous training and validation performance curves are presented in Fig. S9 (SI).

The training MSE values range from 0.0047 to 0.0084, with an average value of approximately 0.0070. Meanwhile, the validation MSE varies from 0.0045 to 0.0308, yielding an average of approximately 0.0153. The consistently low training errors indicate that the ANN model effectively captures the nonlinear relationships between the input variables (adsorbent dosage, temperature, initial concentration, pH, and contact time) and the adsorption efficiency.

Importantly, the validation errors remain within the same order of magnitude as the training errors, demonstrating that the model maintains good predictive capability when applied to unseen data. The absence of significant divergence between training and validation curves (Fig. S9, (SI)) further confirms that overfitting is effectively avoided.

Although some variability in validation MSE is observed across different folds, this behavior is expected due to the limited dataset size (*n* = 49) and the inherent heterogeneity of adsorption systems. In particular, the slightly higher error observed in certain folds reflects sensitivity to data partitioning rather than model instability, and remains within an acceptable range for small-sample machine learning applications.

Overall, the ANN model demonstrates strong predictive performance and good generalization ability across all cross-validation folds. These results confirm that the model is reliable for predicting adsorption efficiency within the investigated parameter space. The robustness of the ANN model is further supported by the consistency between cross-validation results and experimental trends, confirming that the model captures physically meaningful relationships rather than overfitting noise.

#### 3.6.1. Best architecture

**Fig. 21:**
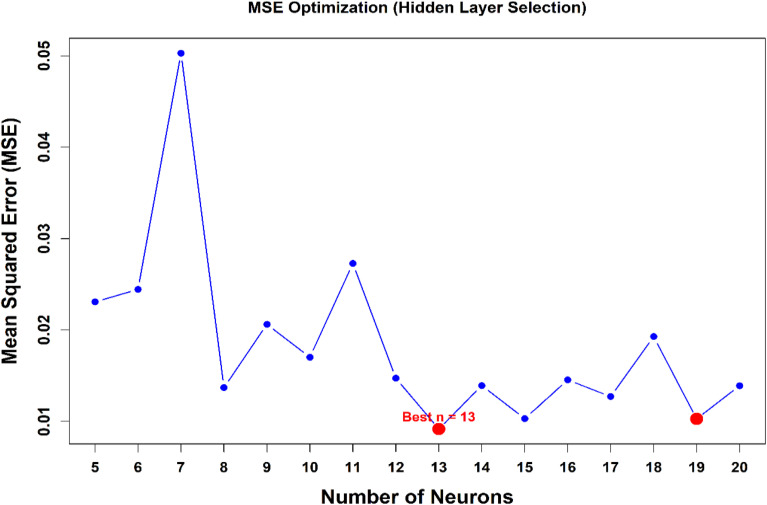
Optimization of the ANN architecture based on the number of hidden neurons *vs.* MSE).

The number of hidden neurons was optimized by varying neurons from 5 to 20 and evaluating the mean squared error (MSE). Results shown in Fig. 21 indicate that MSE varied nonlinearly with neuron number. This trend reflects the trade-off between learning capacity and overfitting.

**Fig. 22:**
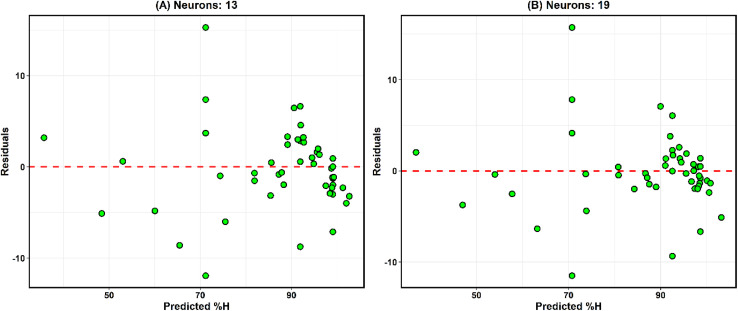
Residual plots of the ANN models with (A) 13 and (B) 19 hidden neurons. The dashed red line represents zero residual.

The highest MSE occurred at *n* = 7 (≈0.050), indicating inadequate feature extraction (underfitting). As the number of neurons increased, MSE decreased significantly and reached the lowest value at *n* = 13 (≈0.009). This configuration was identified as the optimal architecture. Although *n* = 19 also produced a relatively low MSE (∼0.010), it did not outperform *n* = 13 and increased model complexity. Therefore, the ANN with 13 hidden neurons was selected. This architecture balances prediction accuracy and generalization ability. To confirm stability and avoid overfitting, residual analysis was conducted for both *n* = 13 and *n* = 19 architectures (Fig. 22).

#### 3.6.2. Comparison between predicted and experimental data

Model agreement was evaluated using residual plots for the two candidate architectures (Fig. 22). For the 13-neuron model, residuals were symmetrically distributed around the zero line. No increasing or decreasing trend was observed. Most residuals clustered near zero with a narrow dispersion range. This indicates low prediction error and absence of systematic bias. No funnel shape or curvature was detected, suggesting homoscedastic error behavior.

**Fig. 23:**
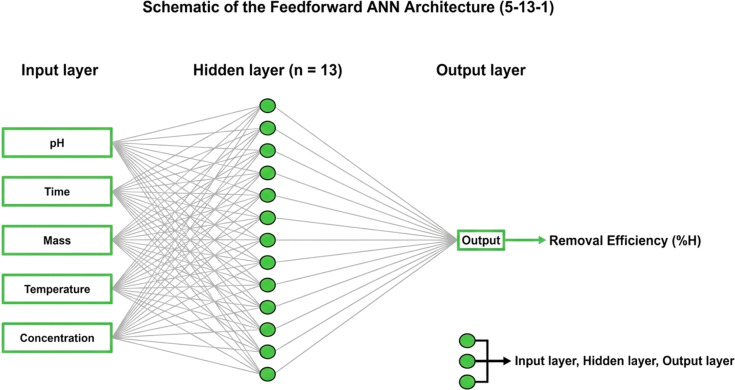
Schematic illustration of the feedforward artificial neural network (ANN) architecture (5–13–1) employed for predicting methylene blue removal efficiency. The network consists of five input neurons (pH, contact time, adsorbent dosage, initial MB concentration, and temperature), one hidden layer with 13 neurons, and one output neuron representing removal efficiency (%).

The 19-neuron model also showed random residual distribution. However, dispersion widened at higher predicted values. Several larger deviations were observed compared with the 13-neuron architecture. Although both models had comparable MSE values, increasing neuron number raised the number of free parameters. With limited data, an overly complex network may learn noise rather than true physicochemical relationships. In contrast, the 13-neuron model achieved the lowest MSE and more stable residual distribution. Therefore, the selection of *n* = 13 follows the parsimony principle. This ensures balance among accuracy, stability, and generalization. The optimized architecture (5–13–1) is illustrated in Fig. 23.

#### 3.6.3. Regression analysis between predicted and experimental data

**Fig. 24:**
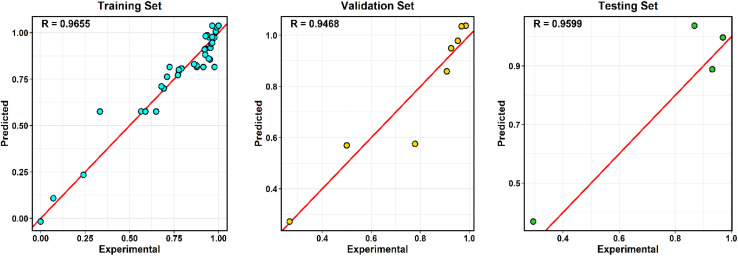
Regression plots of predicted *versus* experimental values for the optimized ANN model (13 hidden neurons) on the training, validation, and testing datasets.

After selecting *n* = 13 as optimal, regression analysis was performed to verify generalization ability (Fig. 24). Predicted and experimental values showed strong linear agreement across training, validation, and testing sets. Data points closely followed the *y* = *x* line, indicating high predictive consistency.

The correlation coefficient reached *R* = 0.9655 for the training set. For validation and testing sets, *R* values were 0.9468 and 0.9599, respectively. The small differences among datasets confirm the absence of significant overfitting. No abnormal dispersion or systematic deviation was observed. The testing *R* value was comparable to the training value, indicating that the model learned nonlinear relationships rather than memorizing data. This confirms the reliability of the ANN in predicting new operational conditions.

#### 3.6.4. Sensitivity analysis and mechanistic implications

**Fig. 25:**
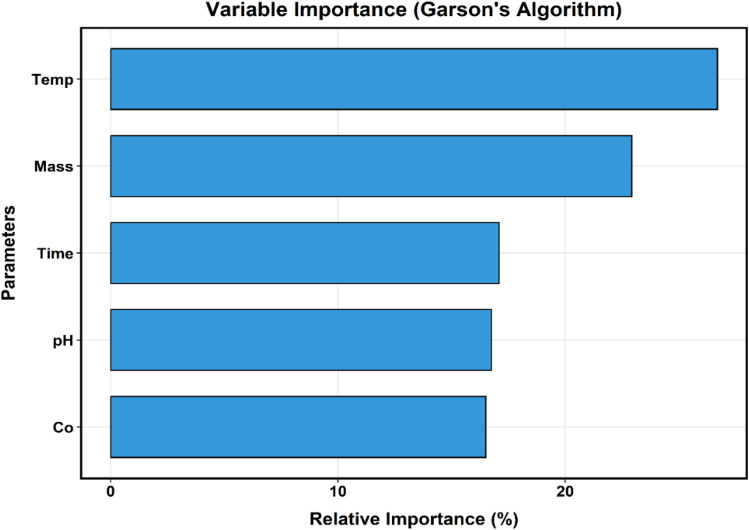
Sensitivity analysis of the ANN model: Relative importance (RI%) of various operational parameters (temperature, adsorbent mass, contact time, pH, and initial concentration (*C*_0_)) on the adsorption process based on Garson's algorithm.

To elucidate the physicochemical significance of the optimized ANN model, Garson's algorithm was applied to quantify the relative importance (RI%) of input variables (Fig. 25). The analysis provides a weight-based estimation of how each operational parameter contributes to methylene blue (MB) removal efficiency, thereby enabling mechanistic interpretation beyond predictive performance.

Temperature exhibited the highest influence (RI ≈ 27%), identifying it as the dominant governing parameter. This strong thermal dependence is consistent with thermodynamic results showing a positive Δ*H*°, confirming that the adsorption process is endothermic. Elevated temperature likely enhances molecular mobility, reduces intraparticle diffusion resistance, and facilitates activation of interfacial adsorption sites within the hierarchical ZnO-carbon framework. The ANN-derived dominance of temperature therefore quantitatively supports the experimentally established thermally activated adsorption mechanism.

Adsorbent dosage ranked second (RI ≈ 23%), reflecting the critical role of accessible surface area and active-site density. Increasing dosage increases the number of available adsorption sites and shortens diffusion pathways, directly influencing removal efficiency. This finding is consistent with BET-derived porosity data and with the Langmuir isotherm behavior observed experimentally, indicating monolayer adsorption on energetically heterogeneous yet finite active sites. The strong statistical weight assigned to dosage further corroborates the importance of interfacial site availability in governing adsorption energetics.

Contact time and pH exhibited comparable influence levels (RI ≈ 16–17%), suggesting cooperative control of adsorption performance by kinetic and surface-charge effects. Contact time primarily governs adsorption kinetics and intraparticle diffusion processes, whereas pH regulates surface charge characteristics relative to the experimentally determined pH_p_zc. At pH values above pH_p_zc, the negatively charged ZnO-ACRPS surface promotes electrostatic attraction toward cationic MB molecules. However, the moderate RI value of pH indicates that electrostatic interaction alone does not dominate the system. Instead, adsorption proceeds through coupled contributions of π–π stacking between aromatic domains, ZnO-mediated Lewis acid–base interactions, pore-filling effects, and diffusion-controlled transport.

Initial concentration displayed slightly lower RI, yet remains mechanistically important as the primary mass-transfer driving force. Higher concentration gradients enhance external diffusion and promote occupation of available adsorption sites, particularly during the rapid initial stage identified in kinetic modeling.

Overall, the consistency between ANN-derived variable importance and independently obtained thermodynamic, kinetic, isotherm, BET, and pH_p_zc analyses suggests that the model captures key factors influencing MB adsorption performance.

However, the ANN results should be interpreted as supportive insights into the relative importance of operational parameters rather than direct evidence of the adsorption mechanism.

#### 3.6.5. Statistical indicators

The predictive performance of the optimized ANN (*n* = 13) was evaluated using *R*, *R*^2^, RMSE, MAE, MAPE, and normalized MSE (Table 6).

**Table 6:** Statistical indicators used to evaluate the predictive performance of the optimized artificial neural network (ANN) model for Methylene blue removal

Metric	Value
*R* (training/validation/test)	0.965/0.947/0.959
*R* ^2^ (test)	0.921
RMSE (%)	5.861
MAE (%)	4.809
MAPE (%)	5.938
Normalized MSE	0.0092

High correlation coefficients were obtained for all datasets. *R* values were 0.965 (training), 0.947 (validation), and 0.959 (testing). The coefficient of determination for the testing set reached *R*^2^ = 0.921. This indicates that 92.1% of the variance in dye removal efficiency is explained by the ANN model. RMSE (5.861) and MAE (4.809) values were low, indicating small average prediction deviations. MAPE was 5.94%, demonstrating high predictive accuracy within acceptable engineering limits. The normalized MSE was 0.0092. This value remained below 0.01, consistent with cross-validation optimization results.

Overall, statistical indicators confirm that the ANN model accurately and stably predicts methylene blue removal efficiency. Compared with conventional linear regression models, the ANN architecture more effectively captures nonlinear interactions and coupled effects among thermodynamic and kinetic variables, which are inherent in heterogeneous adsorption systems.

### 3.7. Proposed adsorption mechanism

To provide a comprehensive understanding of the adsorption process, the mechanism of MB adsorption onto ZnO-ACRPS is interpreted by integrating spectroscopic characterization (FTIR, pH_p_zc, and BET) with non-linear isotherm, kinetic, and thermodynamic analyses.

The isotherm analysis indicates adsorption on energetically heterogeneous surfaces, as reflected by the superior performance of Temkin and Freundlich-type models, suggesting interaction with a distribution of adsorption sites rather than an ideal monolayer.^8^

#### 3.7.1. Surface interactions

Electrostatic interactions are likely significant, as MB exists as a cation (MB^+^) and the adsorbent surface becomes negatively charged above pH_p_zc (7.63). FTIR results show reduced intensity of –COO^−^ and C–O bands, indicating the involvement of oxygen-containing functional groups; however, this evidence remains indirect.^79^

The appearance of aromatic C=C bands (1576–1492 cm^−1^) suggests π–π interactions between MB and graphitic domains of the carbon matrix.^9^ Hydrogen bonding may also contribute, as indicated by the shift of –OH bands, although this interaction is relatively weak.^80^

Changes in Zn–O/Zn–OH bands suggest that ZnO sites may contribute to adsorption as secondary interactions.^81^ Possible donor–acceptor interactions may also occur; however, this interpretation remains tentative due to the lack of direct evidence, although recent computational models support such electronic transfers at Zn sites.^82^

#### 3.7.2. Role of porous structure

The high surface area and mesoporous structure facilitate diffusion and accessibility of adsorption sites. Adsorption is better described by heterogeneous models rather than an ideal monolayer, indicating that the porous structure primarily enhances mass transfer rather than acting as a dominant pore-filling mechanism.^8^

#### 3.7.3. Thermodynamic consistency

The predominance of weak interactions inferred from low Δ*G*° values is consistent with adsorption driven mainly by electrostatic and π–π interactions. The intermediate Δ*H*° and *E*_a_ values suggest possible contributions from moderately strong interactions but do not provide definitive evidence of chemisorption.^83^

Overall, MB adsorption on ZnO-ACRPS is a complex process involving multiple concurrent interactions on a heterogeneous surface. The adsorption is mainly associated with electrostatic attraction and π–π interactions, with secondary contributions from hydrogen bonding and ZnO surface interactions; however, the relative contributions of these interactions cannot be conclusively distinguished.

**Fig. 26:**
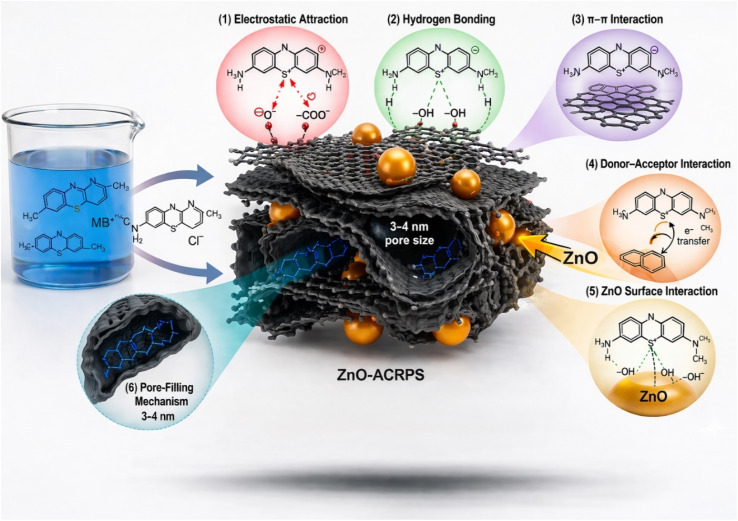
Proposed schematic illustration of the adsorption mechanism of MB molecules onto ZnO-ACRPS.^84^

Fig. 26 presents a schematic depiction of the proposed adsorption mechanism.

The illustration brings together electrostatic attraction, π–π stacking between aromatic domains, hydrogen bonding, donor–acceptor interactions, coordination with ZnO surface sites, and pore-filling within the porous matrix. Each pathway is positioned to reflect its role at the solid–liquid interface, where MB molecules interact with functional groups and active centers on ZnO-ACRPS. The scheme captures the cooperative nature of these surface phenomena and clarifies how concurrent interactions stabilize MB within the carbon–metal oxide framework.

It should be noted that these mechanistic interpretations are based on indirect evidence from FTIR analysis, adsorption modeling, and thermodynamic data. Further studies using advanced spectroscopic techniques (*e.g.*, XPS or *in situ* characterization) are required to provide direct confirmation.

### 3.8. Regeneration and stability

The reusability results of ZnO-ACRPS after methylene blue (MB) adsorption are presented in Fig. S9 (SI). The material maintained considerable adsorption efficiency after three reuse cycles. Specifically, after the first reuse cycle, ZnO-ACRPS achieved 94.35% MB removal. After the second cycle, the efficiency slightly decreased to 93.96%, and after the third cycle, it declined to 50.11%.

Notably, after the first and second cycles, the reduction in MB removal efficiency was negligible. These results indicate that ZnO-ACRPS possesses promising reusability potential for wastewater treatment, thereby contributing to cost reduction and minimizing environmental impact compared with the use of fresh adsorbent materials.

**Fig. 27:**
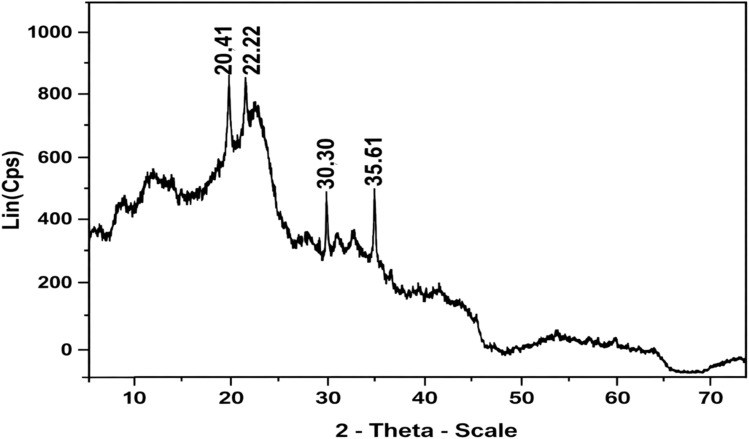
XRD pattern of ZnO-ACRPS after three reuse cycles.

The XRD patterns of ZnO-ACRPS after three reuse cycles (Fig. 27) show that the carbon framework structure remained clearly preserved. This is evidenced by the broad diffraction peaks at 2*θ* ≈ 20.41° and 22.22°, corresponding to the imperfect stacking of turbostratic carbon layers. This structural stability indicates that the carbon matrix did not undergo significant structural collapse during the adsorption–desorption cycles.

The weak signals observed around 2*θ* ≈ 30.30° and 35.61° may be associated with finely dispersed Zn–O domains or poorly crystalline ZnO. These features may result from surface coverage effects, interactions with MB molecules, and small crystallite size rather than structural collapse. The attenuation or broadening of ZnO peaks without loss of the carbon framework has been reported in ZnO/carbon composites after repeated reuse cycles, confirming structural stability despite gradual performance decline.^85^ The significant decrease in MB removal efficiency to 50.11% after the third adsorption–desorption cycle can be explained by several closely related physical and chemical mechanisms.^86^

First, incomplete desorption of dye molecules using 70% ethanol may lead to progressive accumulation of residual MB within the mesoporous framework of the adsorbent, commonly referred to as the “heel” formation phenomenon.^86,87^ This process blocks accessible active sites, reduces the effective surface area, and causes gradual site saturation.^49,87^ Residual dye molecules strongly retained within pore channels significantly increase internal diffusion resistance, thereby limiting MB uptake in subsequent cycles.^86,87^

Second, the partial decline of ZnO nanoparticles (observed as weakened XRD diffraction peaks) suggests surface coverage, structural alteration, or possible leaching during repeated use.^86^ Previous studies indicate that prolonged exposure to dye solutions may reduce the density of active metal oxide sites responsible for electrostatic attraction and surface complexation with cationic dyes.^86,88^ The loss of these active centers directly decreases the overall adsorption capacity.^86^

Finally, repeated washing and drying cycles may induce structural fatigue in the porous carbon matrix.^86,89^ Regeneration cycles can cause partial pore collapse, loss of micro/mesoporosity, and alteration of surface functional groups, thereby increasing mass transfer resistance and hindering intraparticle diffusion.^49,86,87^ Since diffusion-controlled transport was identified as a key kinetic step, any structural deterioration of the pore network significantly impairs dye removal performance.^86,90^

Overall, the combined effects of residual dye accumulation, partial ZnO deactivation, and structural fatigue explain the gradual decline in adsorption efficiency observed after multiple regeneration cycles.^86^

From a practical perspective, the management of spent adsorbent is an important consideration. Although the carbon framework of ZnO-ACRPS remained structurally stable after adsorption cycles, the observed decline in performance indicates partial saturation of active sites. This suggests that regeneration strategies, such as thermal treatment or solvent-assisted desorption, may be required to restore adsorption capacity.

### 3.9. Economic feasibility analysis

To further demonstrate the practical applicability of ZnO-ACRPS, a detailed cost analysis was performed based on a semi-quantitative economic model adapted from recent literature.

The total production cost (*C*_total) was calculated as:*C*_total_ = *C*_raw_ + *C*_chem_ + *C*_energy_where *C*_raw_ represents biomass cost, *C*_chem_ corresponds to chemical reagents, *C*_energy_ accounts for thermal energy consumption, and *C*_misc_ includes auxiliary costs such as washing and gas supply. The estimated cost components are summarized as follows:

• Biomass (rambutan peel and seed): ∼0.0 USD per kg.

• Chemical reagents (H_3_PO_4_ and Zn(NO_3_)_2_): ∼2.7 USD per kg.

• Energy consumption (hydrothermal + pyrolysis): ∼0.5 USD per kg.

• Miscellaneous costs: ∼0.2 USD per kg.

Thus, the total production cost was estimated to be approximately 3.4 USD per kg.

To evaluate the economic efficiency in pollutant removal, a cost-efficiency parameter (Cost_eff_) was calculated: Cost_eff_ = *C*_total_/(*q*_max_ × *η*)

Using the non-linear adsorption capacity, *q*_max_ = 82.95 mg g^−1^ and *η* ≈ 0.95, the effective adsorption capacity corresponds to approximately 78.80 g of contaminant per kg of adsorbent. Accordingly, the cost of pollutant removal was estimated to be approximately 43.2 USD per kg of contaminant removed.

Compared with conventional activated carbons and ZnO-based nanomaterials reported in the literature, ZnO-ACRPS exhibits a significantly lower production cost while maintaining competitive adsorption performance. This is mainly attributed to: (i) the use of low-cost agricultural waste as precursor, (ii) moderate synthesis temperature (400 °C), (iii) simple preparation process without expensive templates or multi-step functionalization.

Therefore, ZnO-ACRPS offers a favorable balance between cost and performance, highlighting its strong potential for large-scale wastewater treatment applications.

The following table provides a comparative assessment of ZnO-and other reported adsorbent materials, focusing on production cost, maximum adsorption capacity (*q*_max_), and overall cost-effectiveness in practical applications (Table 7).

**Table 7:** Cost, adsorption capacity, and cost-efficiency comparison of various adsorbent materials

Material category	Material	Cost (USD per kg)	*q*_max_ (mg g^−1^)	Cost-efficiency	Ref.
ZnO-based	ZnO-ACRPS	∼3.4	82.95	43.2 USD per kg (MB pollutant removed)	This study
Raw biomass	Tiger nut residue	0.01	146.0	0.0219 USD per mol (minimal production cost)	91
	Tomato seeds	118.0	36.23	2137.22 USD per mol (minimal production cost)	91
	Groundnut shell	0.135	∼159.4^a^	0.213 $ per mol (Pb^2+^) (low cost)	92
Biochar	Cherry seed biochar	41.9	94.48	91.80 $ per mol	91
	Rice straw biochar	2.6	35.71	4.76 $ per mol	91
Activated carbon (AC)	Honeydew peel-derived AC	0.26	SBET: 1272 m^2^ g^−1^	Highly cost-effective	93
	Almond shell AC	1.54–2.82	SBET: 822–1458	More competitive than fossil-based AC	94
Magnetic materials	MNCDES (magnetic solvent-based material)	0.0018	87.72	0.205 $ per kg (pollutant)	95
	ZnO–Fe_3_O_4_	1.50	330.0	4.55 $ per kg (pollutant)	95
	MagA (magnetic alunite)	6.70	158.7	42.2 $ per kg (pollutant)	95
	MGO@TNs (magnetic graphene oxide composite)	29.76	322.7	92.2 $ per k*g* (pollutant)	95
Polymer	Chitosan-succinic anhydride	∼25.700^a^	245.0	(Zn^2+^ pollutant) high cost due to high drying energy demand	96
	Chitosan/GLA (cross-linked)	∼9.310	208	(Cu^2+^ pollutant)	96

a The *q*_max_ value of groundnut shell was converted from 0.77 mol kg^−1^ of Pb^2+^ as reported in the literature. The polymer cost was converted from laboratory-scale pricing (€ 21.89 per g) to USD per kg.

ZnO-ACRPS exhibits a relatively low-to-moderate production cost (∼3.4 USD per kg), making it significantly more economical than many advanced nanomaterials or complex modified systems, such as MGO@TNs (29.76 USD per kg).^95^ However, its cost remains higher than that of raw agricultural wastes or low-cost activated carbons derived from inexpensive biomass sources (*e.g.*, melon peel at ∼0.26 USD/kg).^93^ In terms of adsorption performance, ZnO-ACRPS demonstrates a maximum adsorption capacity (*q*_max_) of 82.95 mg g^−1^, which is substantially higher than that of unmodified biochars or raw biomass-derived adsorbents such as rice straw biochar (35.71 mg g^−1^)^91^ and tomato seed-based materials (36.23 mg g^−1^).^91^ Although this value is lower than that of high-performance magnetic composites such as ZnO-Fe_3_O_4_ (330 mg g^−1^)^95^ or MGO@TNs (322.7 mg g^−1^),^95^ ZnO-ACRPS still offers a competitive adsorption capacity within the category of waste-derived adsorbents. More importantly, from a cost-effectiveness perspective, the estimated value of 43.2 USD per kg of MB pollutant removed highlights a favorable balance between economic and technical performance. This advantage is primarily attributed to the utilization of near-zero-cost biomass feedstocks and relatively mild synthesis conditions (400 °C), which significantly reduce energy consumption and overall production cost. Compared with high-cost materials such as modified chitosan-based adsorbents,^96^ ZnO-ACRPS provides a far more economically viable alternative due to its simple preparation route and the absence of expensive templates or multi-step modification processes, thereby enhancing its potential for large-scale wastewater treatment applications. It should be noted, however, that the above comparisons are only indicative, as the reported materials were evaluated for different target pollutants under varying experimental conditions; therefore, a direct and rigorous comparison of adsorption performance and cost-effectiveness is not strictly applicable.

### 3.10. Environmental and practical implications

This study highlights an effective valorization strategy for rambutan peel and seed residues through their conversion into a ZnO-modified activated carbon composite (ZnO-ACRPS). Transforming lignocellulosic agro-waste into a functional adsorbent supports circular economy principles by reducing solid waste while generating value-added materials for wastewater remediation. The obtained turbostratic carbon exhibited a high specific surface area (575.81 m^2^ g^−1^), and ZnO nano-anchoring (10–40 nm) introduced additional active sites and tuned the surface charge (pH_pzc_ = 7.63), resulting in enhanced adsorption performance without the need for costly synthetic precursors.

The preparation route, involving carbonization, chemical activation, and ZnO impregnation, employs scalable and well-established thermal and wet-chemical processes. The synthesis does not require complex instrumentation. The adsorption performance was achieved under mild conditions (pH ≈ 7, 25 °C, 120 min), which may be advantageous for practical implementation. However, further validation under realistic conditions is required. The optimized dosage (20 g L^−1^) and near-neutral operating pH further suggest potential applicability in real wastewater systems, pending validation under complex matrices. Notably, the system requires minimal chemical adjustment. Although the maximum adsorption capacity (96.15 mg g^−1^) is moderate compared to advanced nanomaterials, the removal efficiency (>98%) at relevant concentrations demonstrates competitive performance among ZnO-modified biochar systems.

Isotherm analysis (Langmuir and Temkin) suggests monolayer adsorption on heterogeneous surfaces, while Dubinin–Radushkevich fitting indicates that physisorption and micropore filling dominate, supported by π–π interactions and hydrogen bonding. The amphoteric surface enables effective removal of cationic dyes across a practical pH range.

Overall, ZnO-ACRPS represents a sustainable and scalable hybrid adsorbent integrating biomass-derived carbon and semiconductor oxide functionalities, with potential for cost-effective dye removal, although further studies on long-term stability and real wastewater performance are required.

### 3.11. Limitations and future perspectives

Although ZnO-ACRPS exhibited promising adsorption performance, several limitations should be noted. The adsorption experiments were conducted using methylene blue as a single model pollutant, whereas real textile effluents contain complex mixtures of dyes, salts, surfactants, and competing ions that may significantly affect adsorption behavior. In addition, regeneration and long-term reusability were not systematically evaluated, and the structural stability of the composite under repeated adsorption–desorption cycles remains to be verified. In particular, although high removal efficiency was maintained during the first two cycles, a significant decline was observed in the third cycle, indicating limited durability under repeated use. Mechanistic interpretation was primarily based on isotherm modelling and FTIR analysis; more advanced surface-sensitive techniques (*e.g.*, XPS) would enable quantitative elucidation of post-adsorption chemical states. In addition, the ZnO loading was estimated based on precursor ratios and supported by semi-quantitative EDS analysis. More accurate quantification and chemical state determination using techniques such as ICP, XPS, or TGA would enable a more rigorous correlation between ZnO content, surface properties, and adsorption performance.

Furthermore, although the maximum adsorption capacity (96.15 mg g^−1^) is competitive among ZnO-modified biochar systems, it remains lower than that of some highly engineered nanocomposites, suggesting room for further surface optimization.

Future work should therefore focus on cyclic regeneration studies to assess economic feasibility and durability, as well as validation in real textile wastewater matrices under high ionic strength and competitive adsorption conditions. The semiconductor nature of ZnO also offers opportunities for integrating adsorption with photocatalytic degradation under UV or solar irradiation, enabling simultaneous pollutant capture and mineralization. Surface engineering strategies, including tuning ZnO loading, defect modulation, or heteroatom doping, may further enhance active site density and adsorption energetics. In parallel, process modelling and artificial neural network (ANN) optimisation could support parameter prediction and scale-up design, while life cycle and techno-economic assessments are necessary to evaluate large-scale implementation potential.

Overall, this study demonstrates that ZnO-ACRPS is a sustainable biomass-derived hybrid adsorbent with favourable structural and surface properties for cationic dye removal, warranting further investigation towards potential wastewater treatment applications.

## 4. Conclusions

A ZnO-decorated activated carbon composite derived from rambutan peel and seed was developed, enabling controlled interfacial structuring and enhanced adsorption performance toward methylene blue. The hydrothermal–pyrolytic route facilitated uniform dispersion of wurtzite ZnO within a turbostratic carbon matrix, generating a hierarchical porous structure with accessible active sites.

MB adsorption proceeded *via* a multistep mechanism involving external mass transfer, intraparticle diffusion, and energetically heterogeneous surface interactions. The negative Gibbs free energy and positive enthalpy indicate a spontaneous and endothermic process, corresponding to adsorption favored at elevated temperatures. The adsorption mechanism is dominated by electrostatic attraction and π–π interactions, with additional contributions from ZnO–carbon interfacial coupling through Lewis acid–base interactions.

Regeneration tests revealed a significant decline in adsorption efficiency after repeated cycles, while the structural integrity of the carbon framework remained largely preserved. This suggests that performance loss is primarily associated with progressive active-site blockage rather than structural degradation, highlighting the need for improved regeneration strategies. Future work should also address regeneration efficiency and safe end-of-life management of the adsorbent to ensure practical applicability under real wastewater conditions.

The ANN model demonstrated strong predictive capability and provided quantitative support for the proposed adsorption mechanism. Sensitivity analysis identified temperature and adsorbent dosage as the dominant controlling factors, in agreement with thermodynamic and kinetic interpretations.

Overall, this study establishes a structure–performance–data correlation framework for ZnO–carbon hybrid systems, offering a sustainable strategy for agro-waste valorization and providing mechanistic insights for the rational design of advanced adsorbents with potential applications in wastewater treatment.

## Author contributions

Tra Huong Do engaged in the conceptualization, methodology, and manuscript preparation. Truong Xuan Vuong contributed significantly to data interpretation and writing and editing manuscript. Thi Nguyet Hua, Thi Hien Lan Nguyen and Manh Nhuong Chu contributed equally to data collection, analysis, and manuscript review. All authors approved the final version of the manuscript.

## Conflicts of interest

The authors declare no conflicts of interest.

## Supplementary Material

RA-016-D6RA01824F-s001

## Data Availability

The data supporting this article have been included as part of the supplementary information (SI). Supplementary information: kinetic parameters of the pseudo-first-order (PFO) and pseudo-second-order (PSO) models for methylene blue adsorption at different initial concentrations (Tables S1 and S2), including experimental and calculated adsorption capacities, rate constants, and correlation coefficients (*R*^2^). The SI also includes the UV-Vis calibration curve for methylene blue quantification (Fig. S1), comparative adsorption performance of the prepared materials (Fig. S2), and the effects of pH, contact time, adsorbent dosage, shaking speed, temperature, and initial dye concentration on adsorption performance (Fig. S3–S8). In addition, the reusability and regeneration efficiency of ZnO-ACRPS over three adsorption–desorption cycles are presented (Fig. S9). See DOI: https://doi.org/10.1039/d6ra01824f.
