# Supplementary material for: ZnO-modified activated carbon derived from rambutan (Nephelium lappaceum) peel and seeds for efficient methylene blue removal: adsorption mechanism and artificial neural network modeling
Source: RSC Adv. 2026 Apr 10;16(20):18422–53. doi: 10.1039/d6ra01824f (PMC13068092; doi:10.1039/d6ra01824f)
Supplement: RA-016-D6RA01824F-s001 [file RA-016-D6RA01824F-s001.pdf]

Supplementary Information (SI) for RSC Advances.
This journal is © The Royal Society of Chemistry 2026

Supplementary Information

Title: ZnO-Modified Activated Carbon Derived from Rambutan *(Nephelium lappaceum)* Peel and Seed for Efficient Methylene Blue Removal: Adsorption Mechanism and Artificial Neural Network Modeling

Table S1. Experimental dataset used for ANN training and validation

Mass (g)	Temp (K)	Co (mg/L)	pH	Time (min)	H (%)
0.01	298	51.22	7	120	53.63
0.02	298	51.22	7	120	73.38
0.03	298	51.22	7	120	86.40
0.04	298	51.22	7	120	95.53
0.05	298	51.22	7	120	98.60
0.06	298	51.22	7	120	98.99
0.07	298	51.22	7	120	99.46
0.05	308	156.10	7	120	59.25
0.05	308	156.10	7	120	74.89
0.05	308	156.10	7	120	78.56
0.05	308	156.10	7	120	86.47
0.05	318	152.20	7	120	83.18
0.05	318	152.20	7	120	92.51
0.05	318	152.20	7	120	94.79
0.05	318	152.20	7	120	98.58
0.05	328	150.70	7	120	91.95
0.05	328	150.70	7	120	97.81
0.05	328	150.70	7	120	99.10
0.05	328	150.70	7	120	99.99
0.05	298	49.10	7	120	97.12
0.05	298	102.30	7	120	97.03
0.05	298	157.70	7	120	81.20
0.05	298	200.00	7	120	69.47
0.05	298	270.00	7	120	56.87
0.05	298	310.50	7	120	55.22
0.05	298	407.30	7	120	43.27
0.05	298	537.50	7	120	38.92
0.05	298	50.50	3	120	87.23
0.05	298	50.50	4	120	94.38
0.05	298	50.50	5	120	95.22
0.05	298	50.50	6	120	95.46
0.05	298	50.50	7	120	96.59
0.05	298	50.50	8	120	96.05
0.05	298	50.50	9	120	95.52
0.05	298	49.90	7	30	86.33
0.05	298	49.90	7	60	96.60
0.05	298	49.90	7	90	97.21
0.05	298	49.90	7	120	97.81
0.05	298	49.90	7	150	98.05
0.05	298	67.50	7	30	82.28
0.05	298	67.50	7	60	92.45
0.05	298	67.50	7	90	95.40
0.05	298	67.50	7	120	97.48
0.05	298	67.50	7	150	98.15
0.05	298	89.50	7	30	80.38

Mass (g)	Temp (K)	Co (mg/L)	pH	Time (min)	H (%)
0.05	298	89.50	7	60	86.05
0.05	298	89.50	7	90	91.58
0.05	298	89.50	7	120	95.84
0.05	298	89.50	7	150	97.82

Table S2. Description and statistical ranges of input and output variables employed in the ANN model. The selected parameters represent the main experimental factors influencing the adsorption process, with removal efficiency (%) considered as the response variable for model training and prediction.

Variable	Symbol	Unit	Min	Max	Description
Adsorbent dosage	m	g	0.01	0.07	Mass of ZnO–ACRPS used in adsorption experiments
Temperature	T	K	298	328	Operating temperature
Initial concentration	C_0	mg/L	49.10	537.50	Initial methylene blue concentration
pH	pH	–	3	9	Initial solution pH
Contact time	t	min	30	150	Adsorption time
Removal efficiency	H	%	38.92	99.99	Output variable (target)

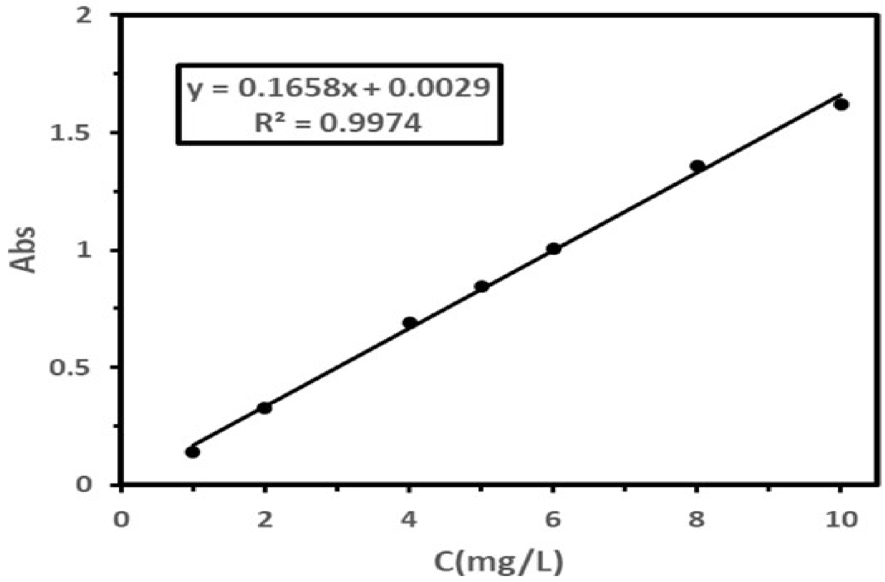

Figure S1. Calibration curve for the quantification of Methylene Blue using UV–Vis spectroscopy

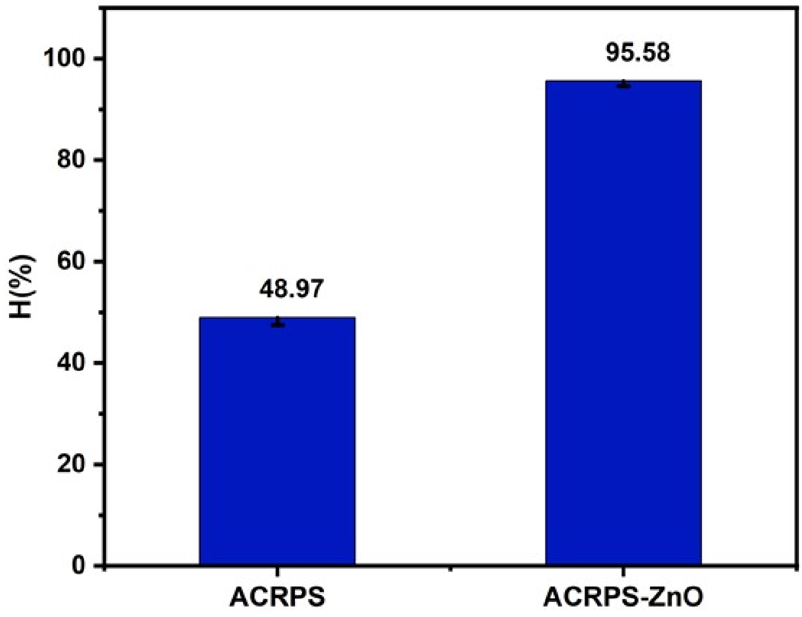

Figure S2. Comparison of the adsorption capacity of two materials (ACRPS and ZnO-ACRPS)

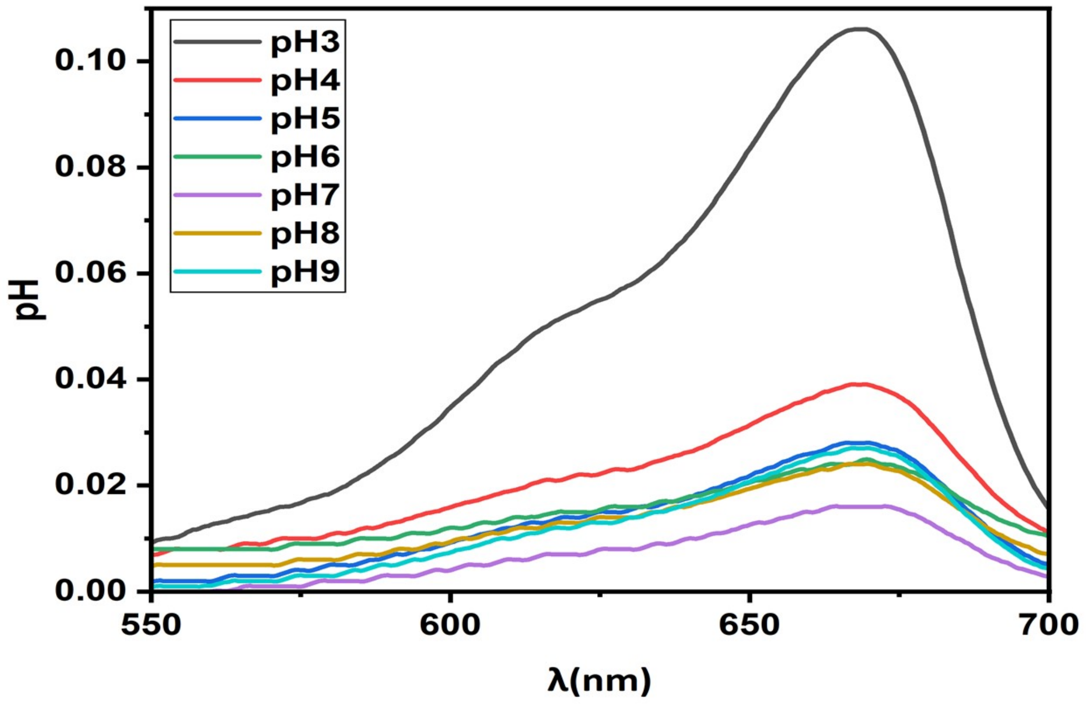

Figure S3. Absorbance as a function of pH.

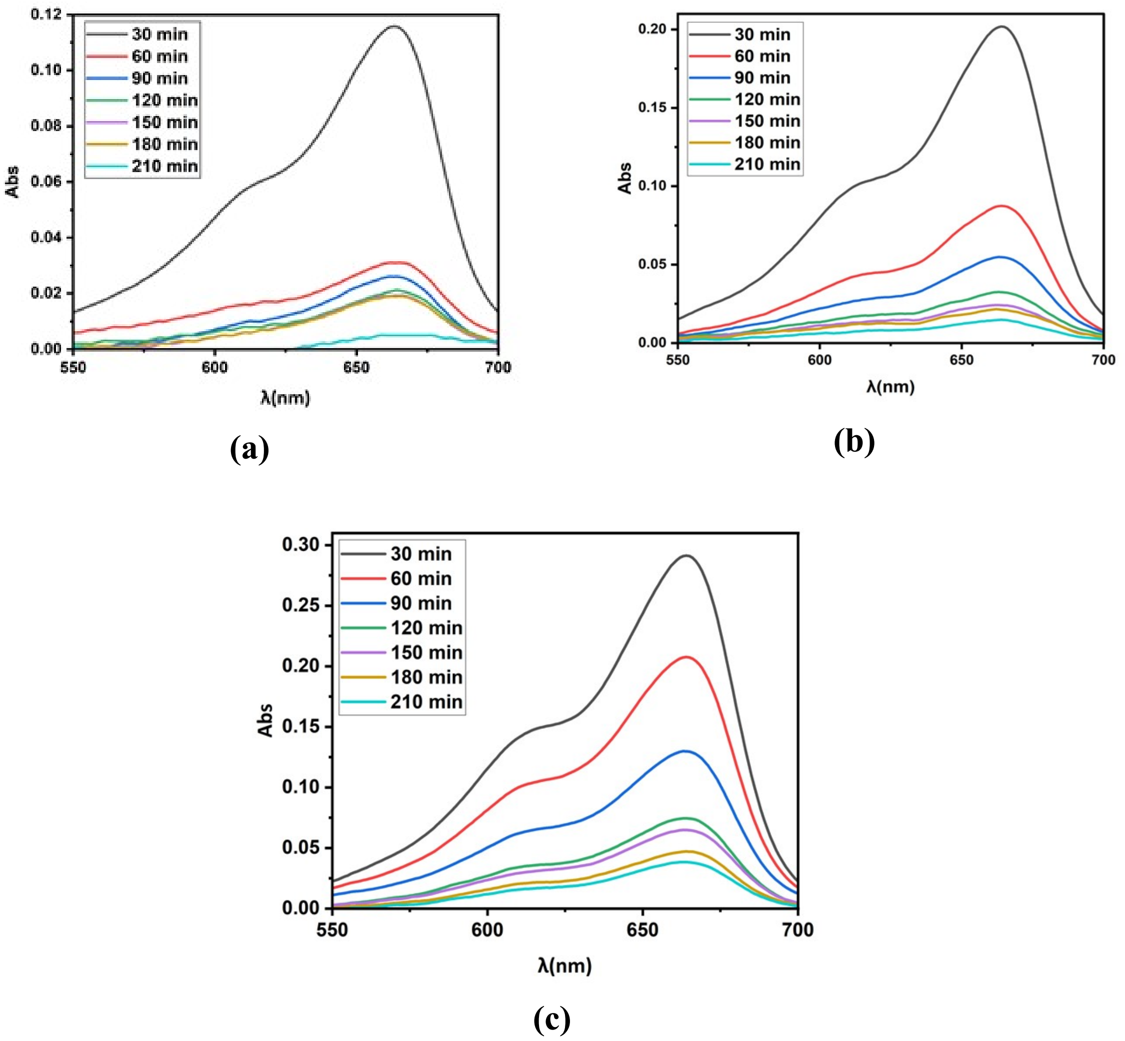

Figure S4. Absorbance as a function of contact time at initial concentrations of 49.89 mg/L (a), 67.46 mg/L (b), and 89.48 mg/L (c).

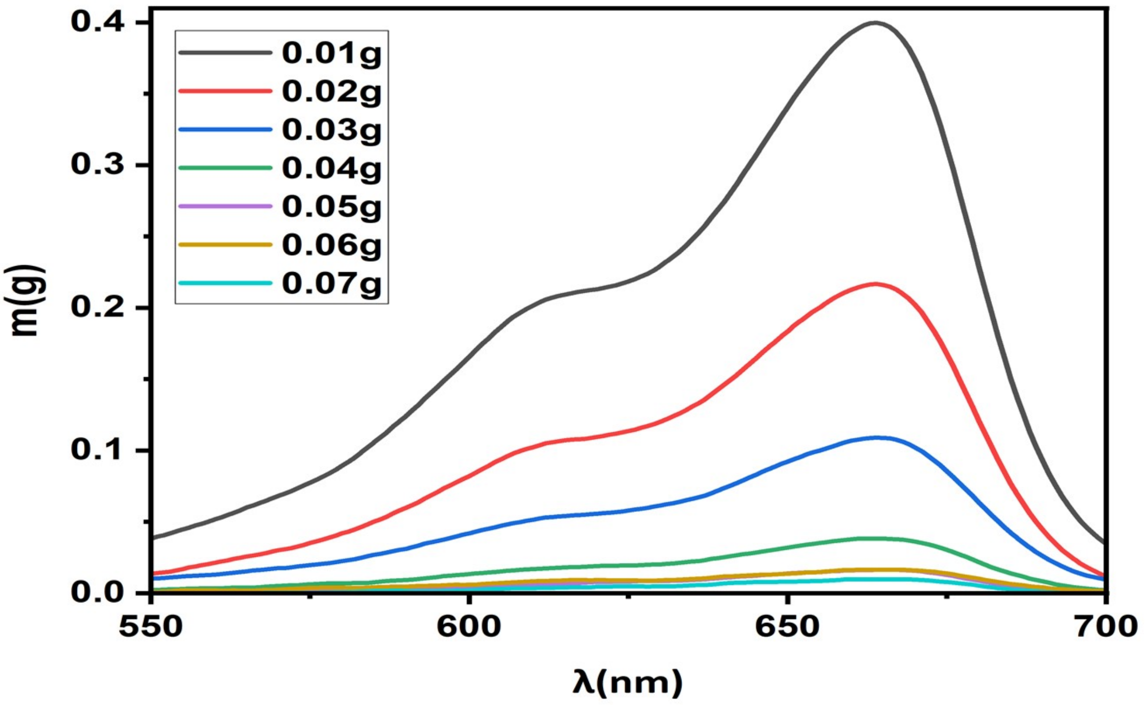

Figure S5. Absorbance as a function of ZnO-ACRPS dosage.

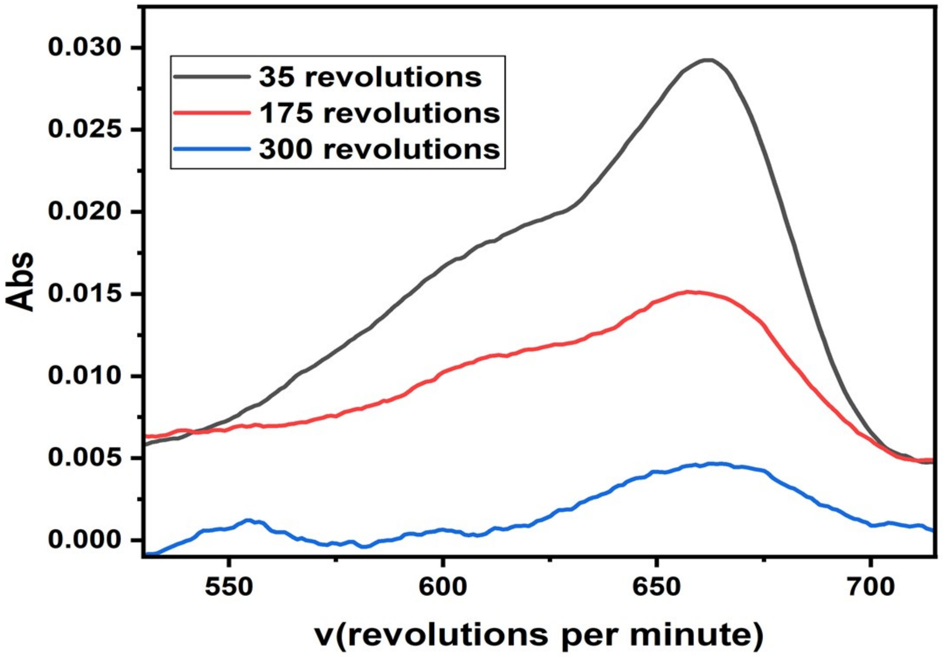

Figure S6. Absorbance as a function of shaking speed.

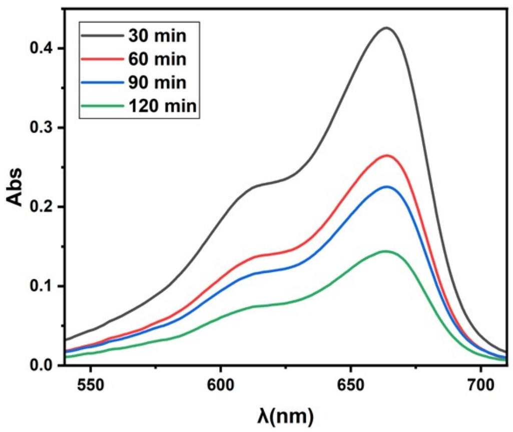

a

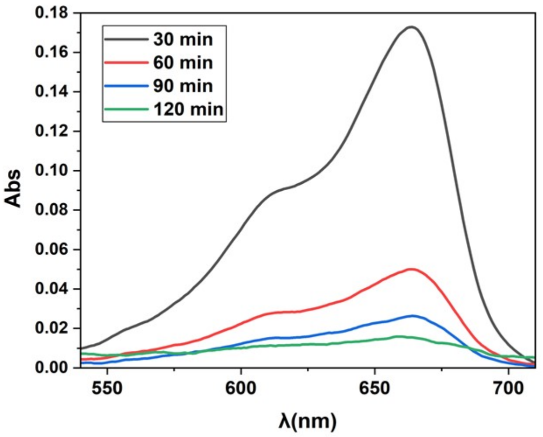

b

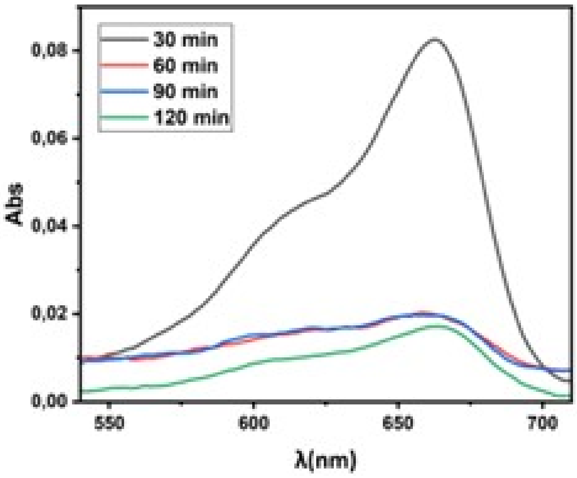

c

Figure S7. Absorbance as a function of temperature: 308 K (a); 318 K (b); 328 K (c).

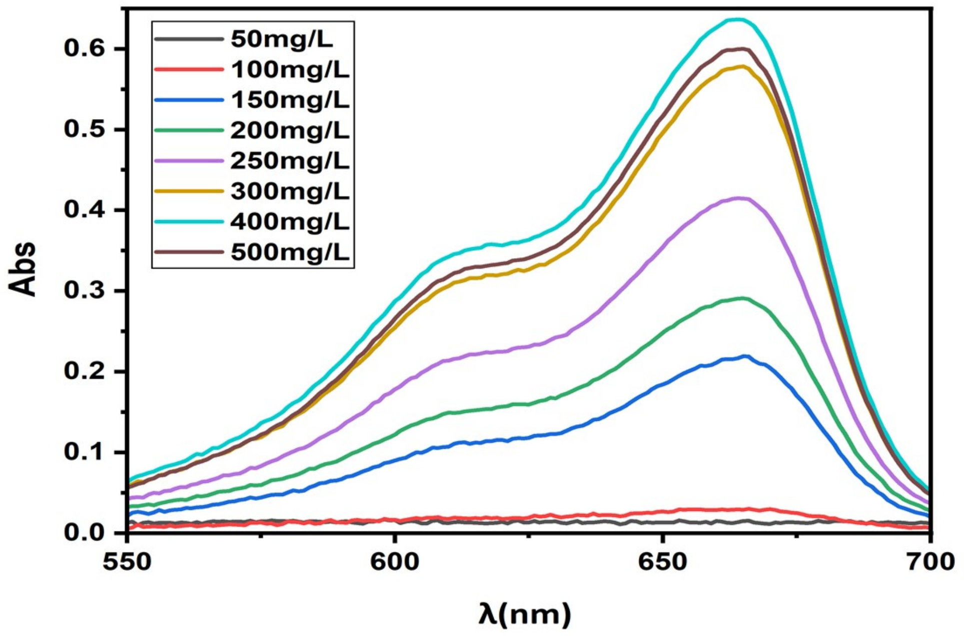

Figure S8. Absorbance as a function of initial concentration

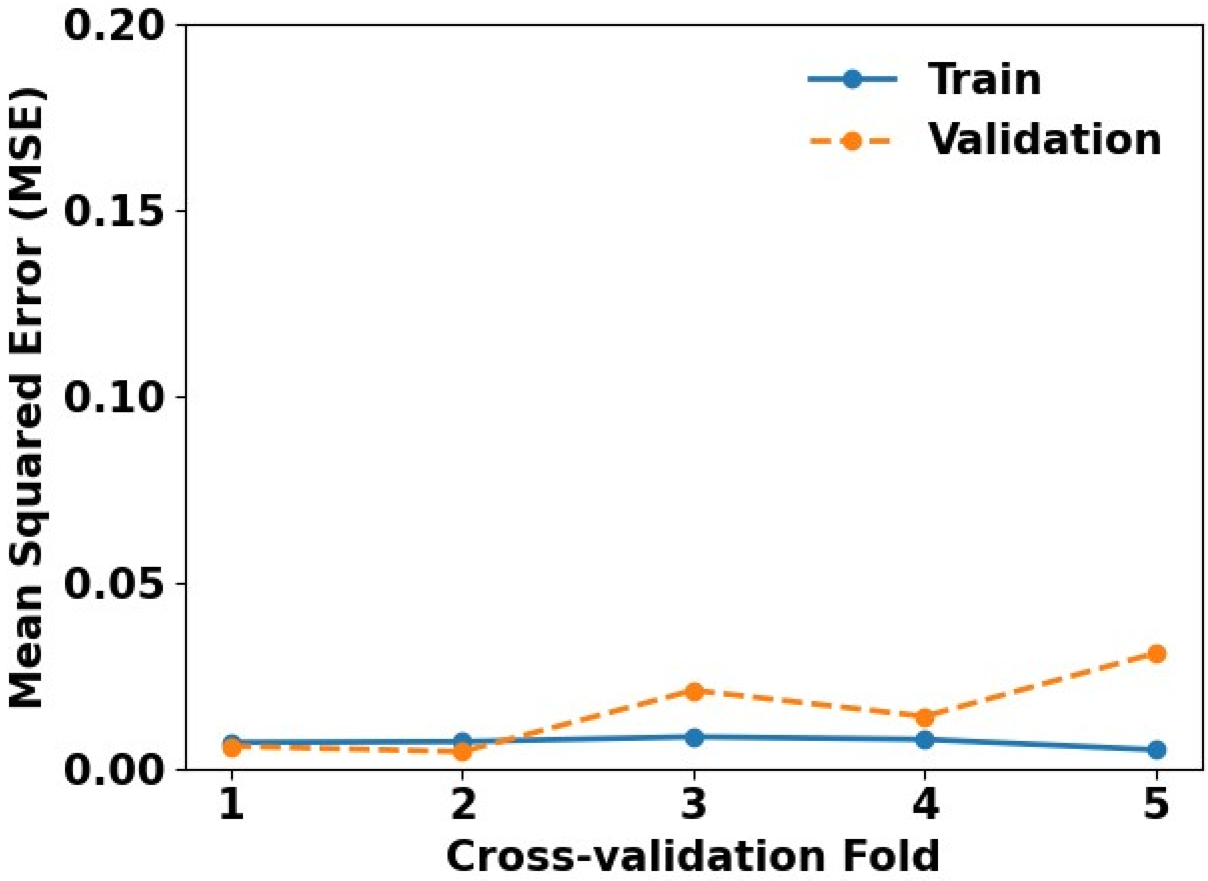

Fig. S9. Cross-validation performance of the ANN model showing the variation of mean squared error (MSE) for training and validation datasets across five folds. The comparable magnitude and consistent trends of training and validation errors demonstrate the robustness and generalization ability of the model, while the absence of

pronounced divergence confirms that overfitting is effectively minimized despite the limited dataset size.

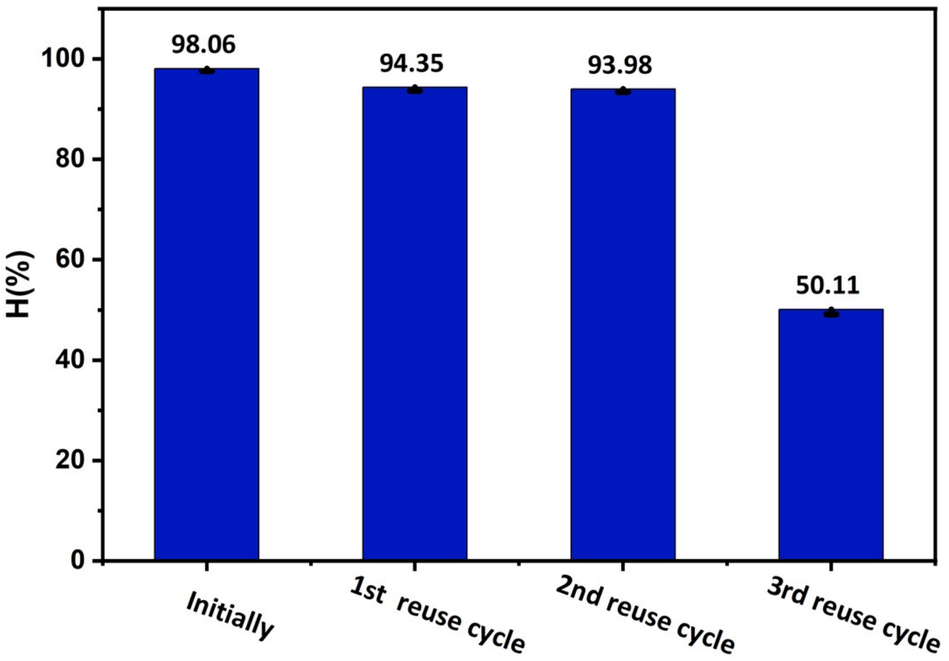

Figure S10. Reusability and regeneration efficiency of ZnO-ACRPS for methylene blue removal over three consecutive cycles.
